# Present state and issues in IORT Physics

**DOI:** 10.1186/s13014-016-0754-z

**Published:** 2017-01-27

**Authors:** Frank W. Hensley

**Affiliations:** 1 Department of Radiation Oncology, University Hospital of Heidelberg, Im Neuenheimer Feld 400, 69120 Heidelberg, Germany; 2 Present address: Birkenweg 35, 69221 Dossenheim, Germany

## Abstract

Literature was reviewed to assess the physical aspects governing the present and emerging technologies used in intraoperative radiation therapy (IORT). Three major technologies were identified: treatment with electrons, treatment with external generators of kV X-rays and electronic brachytherapy. Although also used in IORT, literature on brachytherapy with radioactive sources is not systematically reviewed since an extensive own body of specialized literature and reviews exists in this field. A comparison with radioactive sources is made in the use of balloon catheters for partial breast irradiation where these are applied in almost an identical applicator technique as used with kV X-ray sources. The physical constraints of adaption of the dose distribution to the extended target in breast IORT are compared. Concerning further physical issues, the literature on radiation protection, commissioning, calibration, quality assurance (QA) and in-vivo dosimetry of the three technologies was reviewed. Several issues were found in the calibration and the use of dosimetry detectors and phantoms for low energy X-rays which require further investigation. The uncertainties in the different steps of dose determination were estimated, leading to an estimated total uncertainty of around 10-15% for IORT procedures. The dose inhomogeneity caused by the prescription of electrons at 90% and by the steep dose gradient of kV X-rays causes additional deviations from prescription dose which must be considered in the assessment of dose response in IORT.

**Electronic supplementary material:**

The online version of this article (doi:10.1186/s13014-016-0754-z) contains supplementary material, which is available to authorized users.

## Background

The duty of physics in radiation therapy is to ensure therapeutic quality by providing state of the art technical equipment and procedures, maintaining a safe application of radiation for patients, personnel and environment and by minimizing uncertainties in the therapeutic procedures. Intraoperative radiation therapy (IORT) overcomes many of the technical difficulties by applying radiation directly to the surgically opened tumor bed without irradiating healthy tissue in front of the target. Dose to tissues behind the target is minimized by applying radiation with adjusted penetration such as electrons of appropriate energy or low energy photons (X-rays). Organs of risk within the target area can often be mobilized and removed from the radiation field. Treatment is generally performed with single fields of radiation at a fixed distance between source and target surface which allows minimal treatment planning and usually consists of calculating monitor units for the selected energy and a pre-designed applicator which provides an appropriate dose distribution. Dose distributions in water are generally documented in an atlas for a set of applicators and monitor setting is pre-measured for each applicator and energy.

Despite this very elementary approach of IORT, a large number of physical preparations and quality assurance measures are needed and must be adjusted to the workflow of each individual IORT facility. The majority of these physics issues has been discussed in three reports and guidelines by the America Association of Physicists in Medicine (AAPM) [1, 2] and the Italian Istituto Superiore di Sanità [3], and shall only briefly be enumerated here. The following article reviews publications on the assessment of physical parameters needed to assure treatment quality. A special focus is placed on the uncertainties in these parameters. The review intends to identify issues that presently remain to be solved and to address pathways to their solution, in part also pathways that have not yet been treated in literature. A number of different technologies are used for IORT delivery which are often developed and marketed by a single manufacturer. In the review, the different devices therefore are often identified by their brand names in order to characterize the underlying technology. This naming intends no preference or endorsement for any particular commercial brand and does not imply that a named brand is necessarily superior for the described application.

## Main Text

### Interdisciplinary organization and procedures

The basic roles and interactions of the IORT team members, including surgeons, radiation oncologists, radiation physicists, anesthesiologists, nursing staff, pathologist, and radiation therapist (i.e., the radiotherapy technologist) have been described in the three IORT reports [1–3]. It is essential that each member of the interdisciplinary team knows and respects the responsibilities of the other, often unfamiliar, co-disciplines. To obtain this interdisciplinary education, physics must clearly point out regulatory requirements and legal and biological dose limitations as well as the therapeutic value of physical quality assurance. The complete team must understand the requirement of comprehensive commissioning and continuing periodic controls, the improvements in dose distribution which can be gained by combined/joint treatment planning through physicist and radiation oncologist, the need for continuing research and development and the time and dose requirements to fulfill all of these procedures. Some IORT techniques require long irradiation times so that special anesthetic procedures must be planned, e.g. remote patient surveillance or in some cases a protected position at which the anesthesiologist can remain in the OR during treatment.

### Equipment

IORT equipment must fulfil the needs of more disciplines than usually considered in radiotherapy. Therefore, in advance of selection of equipment, the planned surgical and radiological procedures and set-ups must be discussed within the complete team. Site visits to operational IORT facilities and test set-ups e.g. in the show rooms of the manufacturers or at other sites (or, if possible, in the planned OR) should be made prior to equipment selection to detect possible limitations of certain components. E. g., despite the mobility of modern IORT accelerators, accelerator positioning for certain surgical set-ups may not be possible due to interference of the accelerator stand with the treatment table. The treatment table itself must be carefully selected in order to provide sufficient table top motions to allow beam positioning (in-plane longitudinal and horizontal as well as tilt motions). Beam set-up for certain irradiations (e.g. in the abdomen) may require positioning the patient’s center of mass at large distance from the table column. Table motions, stability and weight capacity must allow for the planned techniques. In this context, also anesthesia access to the patient must be considered and planned. Modern IORT accelerators generally use beam stoppers to reduce OR shielding. The position of the beam stop in relation to the table should be assessed in advance in order to detect possible interference with the table which may preclude the planned technique. The table must provide all accessories needed both for operation and for irradiation. Space around the operation table must be sufficient for accelerator movement and docking. The position of the idle accelerator before and after irradiation must be planned to avoid interference with surgical work. Attention must be paid to the dimensions and weight of the accelerator to ensure that it can pass through all doors, hallways, elevators etc. on its way into the OR. The height of the OR must be suitable to accommodate the accelerator at the most extended position needed for treatment. Floor loads of the OR and all other rooms, hallways and elevators on the accelerators pathways must be sufficient to carry the accelerator’s weight. Many of these issues are discussed in more detail in the IORT guidelines [1–3].

### Electron accelerators

The first generation of IORT accelerators were usually developed in-house on basis of commercial radiotherapy accelerators, in cooperation with the manufacturers. Today, such developments are hardly possible due to medical device regulations. Laborious certifications must be achieved, and companies are often reluctant to support in-house developments in order to avoid liabilities. Therefore the in-house developments have been almost completely replaced by specialized small mobile IORT accelerators. At present, three commercial IORT linear accelerators (linacs) are available on the market. They are designed as mobile devices in order to be installed in an existing operation room without the need of reconstructions for wall mounting. They allow moving the accelerator to the patient and not vice versa and can so avoid transport of the surgically opened patient.

They are specially designed for light weight so that they do not exceed typical floor loads. The designs are specialized for production of low stray radiation by avoiding materials with high atomic number that would cause increased bremsstrahlung. They have straight beam lines without bending magnets for energy selection and some have special techniques of beam focusing. Both measures minimize interactions of defocused electrons with beam line and accelerator structures and thus bremsstrahlung production. Consequently, the stray X-ray background produced by the mobile accelerators is considerably smaller than that of conventional radiotherapy accelerators [4, 5].

**Fig. 1:**
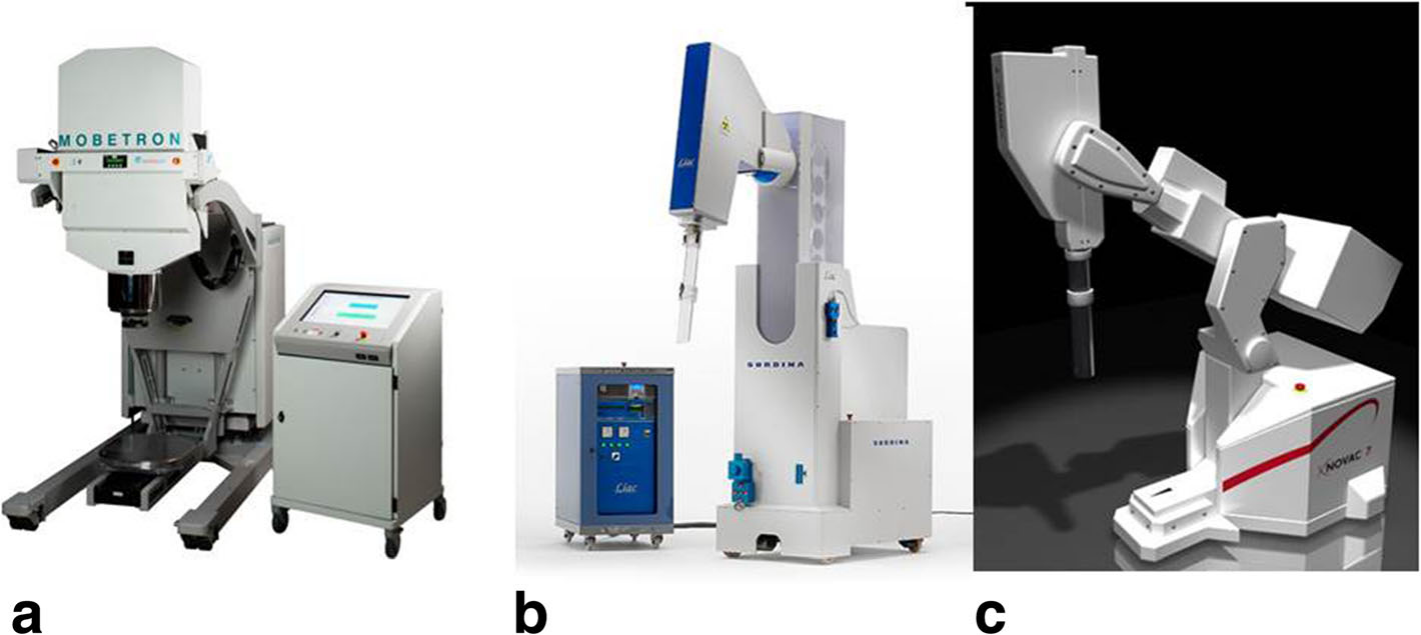
Mobile electron accelerators for intraoperative radiation therapy. **a** The Mobetron (IntraOp Medical Corporation, 570 Del Rey Ave, Sunnyvale, CA 94085, USA), **b** the Liac, **c** the Novac 7 (both: Sit Sordina IORT Technologies Spa, Galleria del Pozzo Rosso, 13, 36100 Vicenza VI, Italy)

The present linacs provide electron beams with energies between 4 and 12 MeV, depending on type. Beam energies typically increase in steps of 2MeV or 3 MeV, equaling an increase in penetration of around 7mm or 1cm per step. Lower penetration or intermediate steps in penetration can readily be achieved by insertion of water equivalent bolus between applicator and patient. Higher energies than 12 MeV are only infrequently offered by the manufacturers to avoid neutron contamination which may cause need for additional shielding. Neutron production by interactions with heavy elements begins at electron energies above 8 MeV for Pb or above 10 MeV for Cu and Fe [5]. For the commercial IORT linacs, measurements by Soriani [4], Loi [6] and Jaradat and Biggs [7] have shown that the neutron doses produced by the IORT linacs are considerably smaller than those for conventional linacs, and that usually no additional shielding for neutrons is needed. Figure 1 shows the three accelerators presently on the market.

#### Applicators

**Fig. 2:**
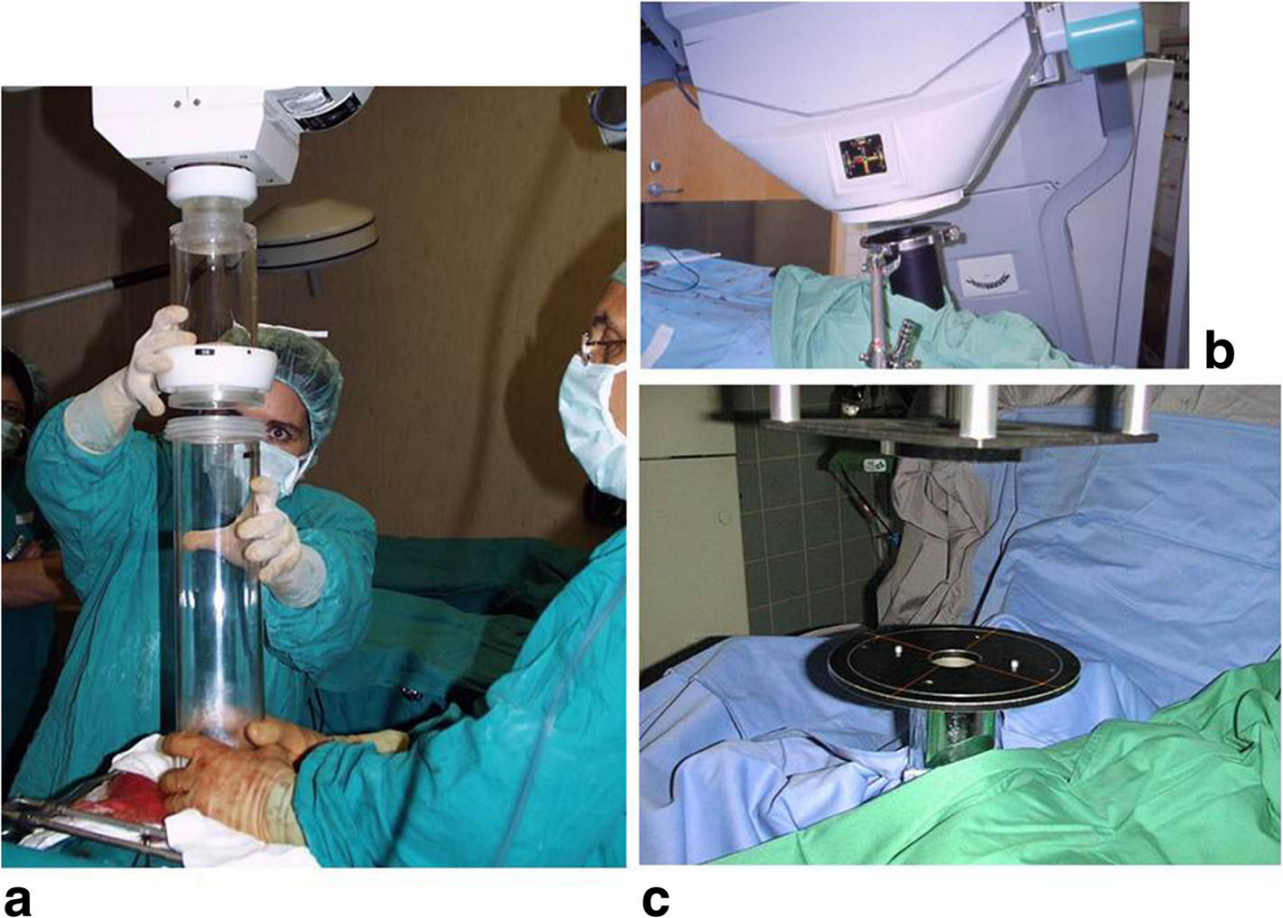
Hard and soft docking technique in electron IORT. **a** Hard docking: the applicator is attached to a receptor on the accelerator head by a sterile person while a second sterile person holds the applicator in place. (shown for a Liac accelerator). **b** Soft docking: the applicator is attached to the couch with a table stand. There is no mechanical connection between applicator and accelerator. (shown for a Mobetron accelerator). **c** The alignment of accelerator and applicator is adjusted with a laser alignment system. (Shown for a Siemens Mevatron ME accelerator)

The electron beam of an IORT accelerator is collimated by a fixed conical primary collimator placed immediately behind the exit window of the accelerator guide. Final collimation is achieved with a set of cylindrical applicators of various diameters in order to produce different field sizes. The sterilized applicator is inserted by the surgeon and radiation oncologist directly into the surgical opening above the target in a position and entrance angle which directs the beam through the underlying target tissues. Alignment of the applicator with the beam is adjusted by moving the accelerator to the correct position and angle, providing an unchanged position of the applicator. This is achieved by two techniques: with hard docking the applicator is mechanically locked onto the accelerator head. This means one must move the accelerator over the end of the applicator and carefully attach the applicator end to a receptor on the head (Fig. 2a) without moving the applicator. With the soft docking technique (Fig. 2b, c) the applicator is rigidly attached to the table with a stand and clamp. The accelerator is also moved over the applicator end but not mechanically attached. Alignment is achieved at distance by a laser guidance system which indicates the correct centering, angle and distance of applicator and accelerator. The principle of soft docking systems is explained in detail in references [2, 8] and [9]. Advantages of this system are the lower risk of injury of the patient by forces from the moving accelerator or electrical contact to the machine. The applicator is fixed in its position so that no misalignment from its intended entrance angle by movement during the docking process can occur. The soft docking system uses thin-walled metallic applicators while the hard docking machines have transparent plastic applicators allowing a direct view of the target during the docking procedure. A disadvantage of the plastic applicators can be their thicker walls which require a larger surgical opening, a disadvantage of hard docking can be longer time for machine alignment.

Depending on the planned treatment sites one may encounter limitations in applicator size and design. Standard (round) applicators are usually provided by the manufacturers in a range between 3-4cm up to 10cm diameter in steps of 1cm or, by some manufacturers, 0.5cm. One manufacturer (Intraop) provides elongated applicators with field sizes of 7×12 cm^2^, 8×15 cm^2^ and 8×20 cm^2^, which use an additional flattening filter which reduces the output by approximately a factor of two and reduces the beam penetration (R_90_) by about 2mm. A prototype of these applicators is described by Janssen et al. [10]. Larger field sizes can be achieved by using applicators with a beveled end, however one must be aware that the dose distribution of such an applicator is asymmetrical, extends into tissue beyond the applicator tip at an angle and has less penetration in depth under the beam entrance surface than the comparable straight applicator. Abutting fields is possible with applicators providing one straight side or by producing a straight field edge with an absorber under the applicator entrance [11, 12]. Characteristics of dose distributions from beveled applicators with and without additional field shaping by straight lead absorbers are described by Esposito et al. [13] and further discussed in the section on commissioning in electron IORT. Applicators with one straight side for field matching (“Squircle applicators”) have been published for older machines, but are not regularly provided by the manufacturers [14].The development of a beam shaper producing rectangular and squircle fields of adjustable size is reported for one of the mobile accelerators [15]. The applicator ends at a distance above the patient surface. Soriani et al. describe the changes in electron depth dose behavior and output factors connected to both field shaping and the distance of the applicator from the patient surface [15]. The correct alignment of this device is not clear. The authors propose a light field which so far has not been described in publication. It must be recognized that any method of field matching requires an examination of the correct gap width between the abutted fields in order to avoid hot or cold spots at the intersection.

Attention must be payed to applicator sterilization workflow so that the required applicator is always available at the time of operation and applicators are not damaged during sterilization. Sterilization of the applicators must be planned together with nursing staff. The number of applicators should allow for doubles of those applicator sizes which are probable to be used twice in one day.

### IORT irradiators using kV X-rays

#### External kilovoltage X-ray generators

**Fig. 3:**
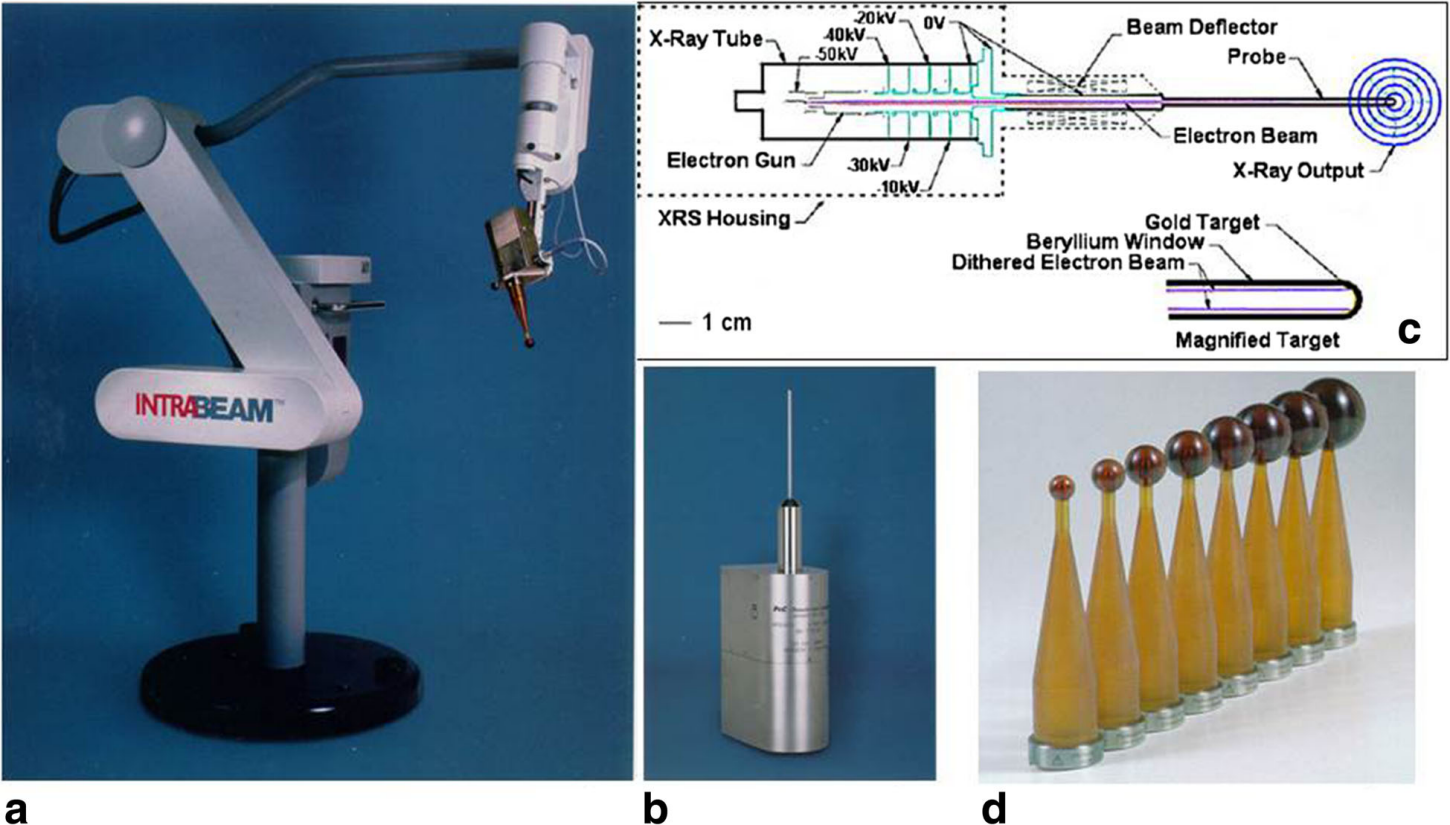
The Zeiss Intrabeam System (Carl Zeiss Meditec AG, Göschwitzer Str. 51-52, 07745 Jena, Germany). **a** The X-ray source (XRS) with a spherical breast applicator mounted on the floor stand. **b** The XRS. **c** Schematic drawing of the XRS accelerator. **d** Spherical applicators for breast IORT

**Fig. 4:**
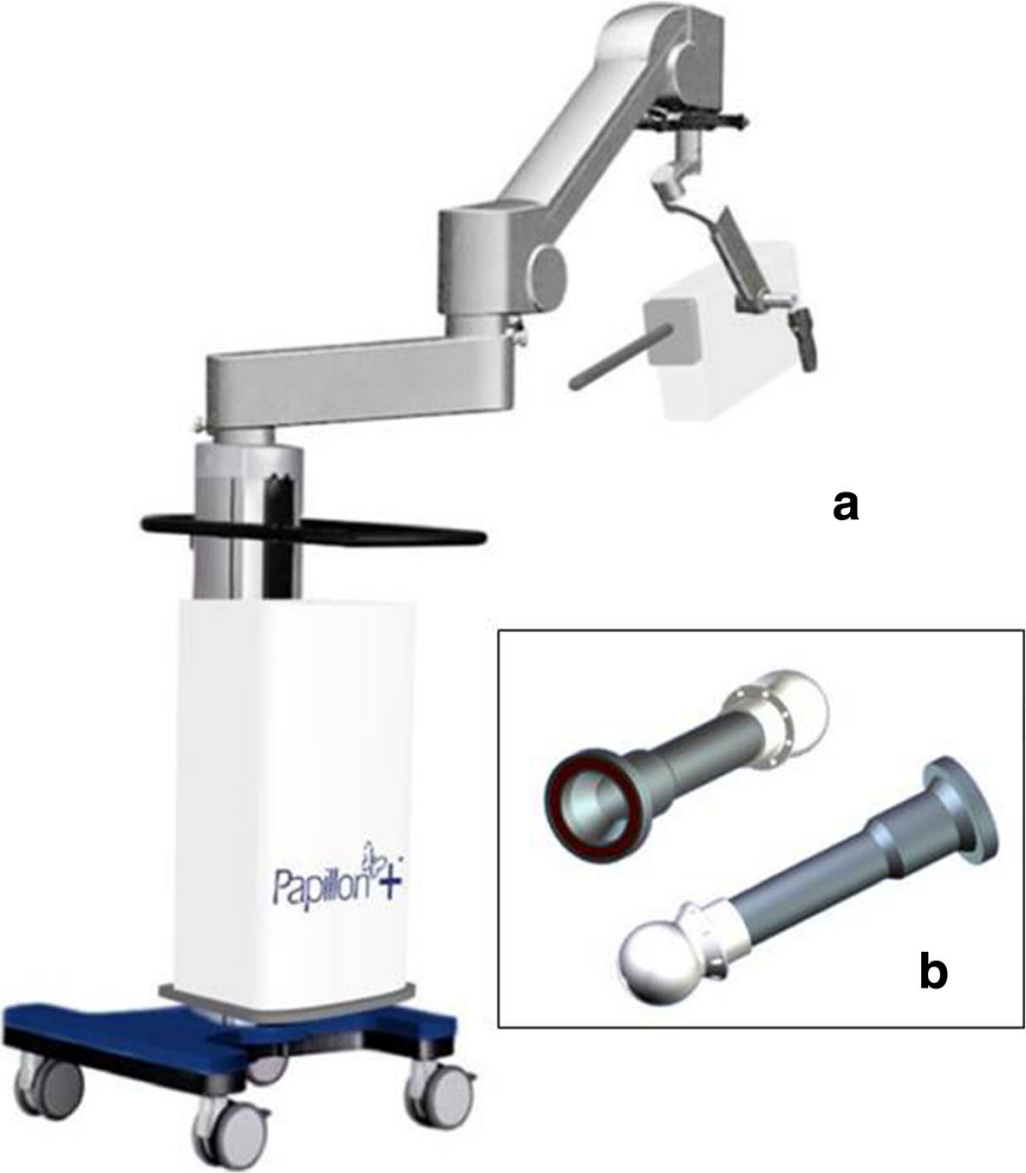
The Papillon system (Ariane Medical Systems Limited, Derby DE1 3BY UK). **a** The X-ray source mounted on the floor stand. **b** Spherical applicators for breast IORT

Several devices are available for IORT applying low energy X-rays. The most frequently used device is the Intrabeam System (Carl Zeiss Meditec AG, Göschwitzer Str. 51-52, 07745 Jena, Germany, Fig. 3a) , a miniature 50 kV X-ray generator which accelerates a beam of electrons beam from an electron gun down a thin (3.2 mm diameter) drift tube (Fig. 3b) at the end of which it hits a thin hemispherical gold target. The target sits inside a hemispherical Be beam-exit window which is covered by a thin film of titanium nitride for biocompatibility. An isotropic distribution of bremsstrahlung is produced by oscillating (“dithering”) the beam around the tube axis in a circular movement with a set of deflector coils. Detailed descriptions of a precursor type of generator, the Photon Radiosurgery System (PRS) (Photoelectron Corporation, Waltham, MA 02154, USA) are given by Dinsmore et al. [16] and Beatty et al. [17]. This system was purchased by Zeiss and with some improvements (which do not change the physical properties of the generated X-Rays) is now the core technology used in the Intrabeam. Many features of the present system are described by Armoogum et al. [18] and Eaton [19]. The device was first introduced for the irradiation of brain tumors [20]. Using the spherical plastic applicators shown in Fig. 3d it is today mainly used for intraoperative breast irradiation within accelerated partial breast treatment (APBI) [21–23] and also advanced boost treatment [24]. With surface applicators [25], flat intraoperative targets can be treated. Special metallic sleeves to guide the drift tube allow intraoperative irradiation during kyphoplastic stabilization of vertebral metastases (Kypho-IORT) [26, 27]. A similar technique of partial breast IORT is possible with the Papillon system (Ariane Medical Systems Limited, Derby DE1 3BY UK ), which uses a switchable 30/50 kV X-ray generator and a Chaoul-type (hollow) rod anode tube covered by spherical applicators (Fig. 4). A technical description of the X-ray tube is given by Croce et al [28]. An advantage of this system is its higher dose rate of around 10Gy/min for most applications [29] which allows shorter treatment times than the 30-50min for 20Gy in breast treatments with the Intrabeam.

A further class of irradiation techniques in intra and perioperative radiotherapy uses the Xoft Axxent miniature X-ray tube or conventional radioactive afterloading sources. Although radiation is often applied in several postoperative fractions, the surgical procedures concerning target preparation and also the radiotherapy issues of target coverage are comparable to IORT. Especially accelerated radiotherapy of breast cancer with intraoperatively placed balloon catheters is often considered as alternative to IOERT. Other techniques using intraoperative flaps and also intraoperatively placed catheters for later irradiation will not be discussed.

#### The Xoft Axxent miniature X-ray source (“electronic brachytherapy”)

**Fig. 5:**
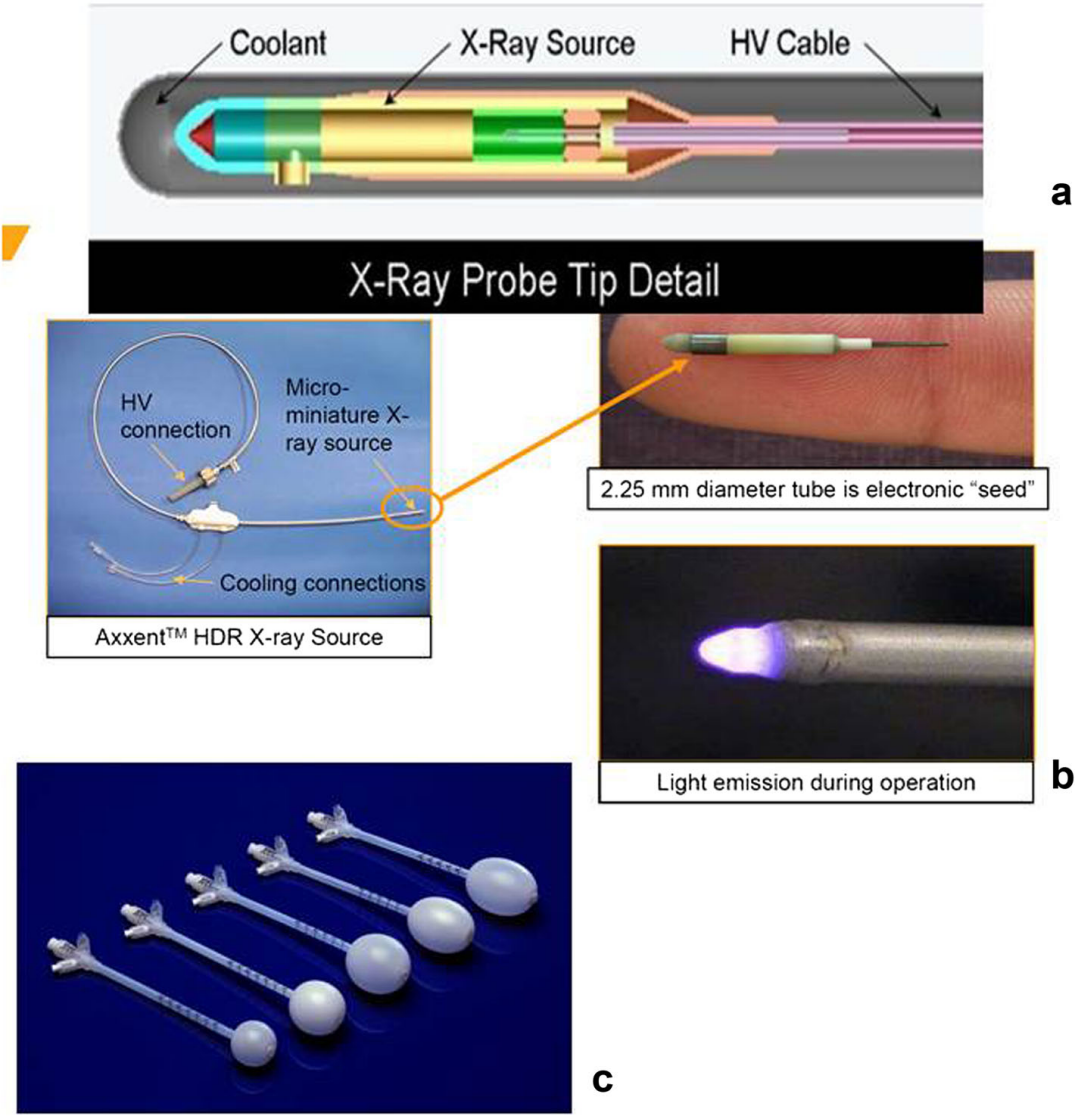
The Xoft Axxent System (Xoft Inc. 345 Potero Ave. Sunnyvale, CA 94085, USA). **a** Schematic diagram of the X-ray source. **b** Left: the cooling tube in which the source is guided to the irradiation position. Right top: The source, bottom: light emission during source operation. **c** Spherical balloon applicators for breast IORT

Due to its small dimensions (15mm length × 2.25mm diameter) the Xoft Axxent source (Xoft Inc. 345 Potero Ave. Sunnyvale, CA 94085, USA, Fig. 5a) can be positioned inside an applicator via a flexible 5.4 mm diameter water-cooling catheter (Fig. 5b). It is used for breast IORT together with a balloon catheter (Fig. 5c). A detailed description of the device and its application is given by Park et al. [30]. Dosimetry parameters for the Xoft Axxent source for dose calculation with the AAPM TG43 formalism [31] are given by Rivard et al. [32] for three voltage settings 40kV, 45kv and 50kV. Originally, the source needed to be replaced after 10 applications (or 170 min of irradiation time) requiring new calibration and quality assurance [30]. Meanwhile the manufacturer’s user manual manufacturer states that 750 min of irradiation time are possible.

The present technology used in kV-IORT is described in a review on electronic brachytherapy by Eaton [33]. Although most of the devices described there are intended for contact brachytherapy, many can also be used for intraoperative irradiation.

The major advantage of kV-IORT is its need for significantly less radiation protection in comparison to high energy accelerators (discussed below). Disadvantages are the steep dose decrease leading to a coverage of only thin layers of target tissue with prescription dose (discussed in the section *IORT targets, target preparation, dose coverage*) together with deeper penetration of radiation and higher doses to tissues beyond the target.

#### Radioactive afterloading sources

The literature on radioactive afterloading sources is not systematically reviewed in this work, however certain aspects are compared since the balloon technique for breast IORT with electronic and radioactive sources is almost identical. Due to the higher photon energies of the afterloading sources (Ir-192 : mean energy 380 keV), conventional afterloading has a slightly flatter dose gradient than kV X-rays (the dose gradient with both modalities is dominated by the inverse square law) but on the other hand requires shielding of the treatment room. Shielding the OR can be avoided by a perioperative approach in which the applicator is placed during surgery, but irradiation takes place at a later time in a shielded brachytherapy vault. For breast treatment with Ir-192 sources, a number of balloon applicators with multiple source guiding catheters (struts) are available which can produce a better target coverage with dose but can also produce higher skin doses. (see paragraph *IORT targets, target preparation, dose coverage*). Different types of balloon applicators are described in a review of APBI techniques by Njeh et al. [34].

### IORT targets, target preparation, dose coverage

Originally the typical target of IORT was the surgical margin in which tumor cells remain due to incomplete resection or microscopic infiltration. The target was therefore usually a superficial thin layer of tissue which can be covered with sufficiently homogeneous dose by low energy electrons or photons. The use of IORT in APBI and boost irradiation within breast conserving treatment of breast cancer has changed this paradigm. The pathological study of Holland et al. showed that in 43% of patients with invasive cancers which are eligible for breast conserving treatment tumor additional tumor foci were found beyond a radius of 2cm surrounding the tumor, the number decreasing to 17% at 3cm and 10% at 4cm [35]. A margin of 1-2 cm of tissue surrounding the original tumor bed is therefore usually taken as target for boost treatment [36]. This requirement defines an extended volume of several cm^3^ which must be prepared for irradiation and covered with the prescription dose. Treating the larger target volume can require higher electron energies or techniques with kV X-rays which both can cause more dose in tissues surrounding the target and in some cases require additional protection of risk tissues.

### IORT with electrons

In IORT with electrons the lateral (side) walls of the excision cavity must be mobilized and drawn together to form a compact target volume which can be completely covered by the applicator. Such a volume will be a few cm thick, so that typically an electron energy is chosen which deposits 90% of the central axis maximum dose at the deep side of the target. Prescription dose is defined at this position so that in effect, a maximum overdose of 11% is accepted in the target [36, 37]. Additionally, since the dose profiles of electron beams are only flat in the central region, the applicator must be selected approximately 2cm larger than the target diameter in order to cover the volume with the reference dose [37]. Many institutions following the original ELIOT protocol protect risk tissues behind the target (ribs, lung, heart) by inserting a shielding plate between the mobilized target and the pectoralis muscle [38, 39]. The shields typically consist of a metal absorber (Pb, CU) covered with a layer of low Z material (Al, PTFE) to absorb backscattered electrons [40, 41]. Possibly, the backscatter can be considered as a desired increase of target dose.

### kV-IORT

**Fig. 6:**
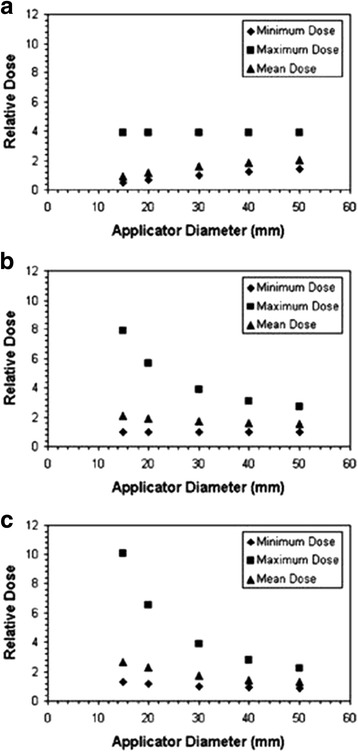
Maximum, mean and minimum doses in 10 mm thick spherical shells of tissue surrounding Intrabeam breast applicators for different depths of prescription of the reference dose with different sizes of applicators. **a** prescription at the applicator surface. **b** prescription at 10mm distance from the applicator surface. **c** prescription at 20mm distance from the applicator surface. The prescription dose is defined as unity for the minimum target dose with the 30mm applicator in each diagram. For small applicators, a prescription at the applicator surface (a) produces smaller overdoses at the applicator surface, however also lower doses at a depth of 10mm which is considered as the margin surrounding the tumor cavity which must be treated. Prescription at 10mm depth ensures that the desired dose arrives at this depth, produces higher overdoses for small applicators, prescription at 20mm depth increases the overdoses. Therefore the authors conclude that a prescription at 10mm depth is the best compromise in order to ensure that desired dose arrives at depth for all applicator sizes. From Ebert and Carruthers (2003) [35]

**Fig. 7:**
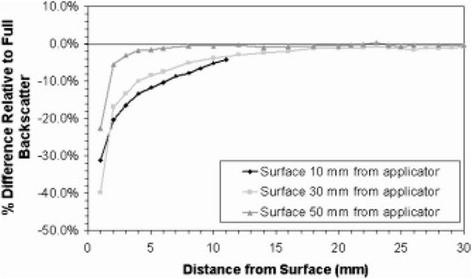
Dose reduction due to missing backscatter at different depths under the tissue surface for three different depths of the applicator surface in tissue (10mm, 30mm and 50mm) for a 30mm diameter intrabeam applicator. For shallow depths of the applicator dose reduction in superficial tissues can amount as much as 25-40%. From Ebert and Carruthers (2003) [35]

With the spherical applicators and balloons used with kV X-rays and Ir-192, target preparation must ensure a close adherence of the surgical cavity to the applicator surface. Tissues at distance will receive significantly lower doses due to the steep dose gradients. The skin must be retracted from the entrance of the applicator to minimize skin dose [21]. With the spherical applicators used with the Intrabeam and Papillon system, dose is usually prescribed at the applicator surface and (largely due to inverse square of distance) decreases to around 28-37% at 10mm distance from the surface, depending on applicator size, and to 11-20% at 20mm distance [42–44]. Skin dose can be a concern when the applicator is close to the surface although a question to be resolved is what the desired maximum dose should be [21]. With these applicators, the typical prescription dose is twice the dose given with electrons resulting in around 46-80% of typical electron doses at 1cm and 22-40% at 2cm depth. Ebert and Carruthers [42] use Monte Carlo simulations to calculate dose-volume histograms (DVHs) for these heterogeneous dose distributions in a target shell of 10mm thickness surrounding different applicator sizes which is considered as the volume needing treatment. From these DVHs, the relation of maximum (surface), mean and minimum dose is calculated for three different methods of dose prescription at either the applicator surface or at 10mm and 20mm target depth (shown in Fig. 6), and compared for different applicator diameters. The comparison shows that the least variation in overdose in the target is achieved with prescription at the surface of different applicator sizes, however at cost of the mean and minimum doses (Fig. 6a). The authors conclude that dose prescription at 10mm depth is probably the best compromise in order to ensure the desired dose at this depth for all sizes of applicators (Fig. 6b). (Note that the Targit trial recommends dose prescription at the applicator surface [21, 22].) The calculations show that the dose decrease of low energy X-rays in breast tissue is steeper than in water. This can lead to an underdose of 3-5% if calculated in water. They also show the higher doses to bone (around a factor 3 for the first mm of bone tissue) and the dose reduction due to missing backscatter when the target is near the skin. Dose reductions can range between 25-40% for tissues at 1 cm from skin and 0-10% for tissues at 5 cm from the skin. The larger reduction at both tissue-skin distances holds for an applicator-skin distance of 10mm, the smaller for a distance of 5cm Fig. 7). Breast treatments are usually performed covering the patient surface over the applicator with a lead-rubber protection sheet similar to those provided by Zeiss (0.25mm Pb equivalent). Using the Ebert and Carruthers data [42] one can estimate that the backscatter from the shielding sheet will compensate for some of the reduction of surface dose. On the other hand backscatter will increase skin dose, however to a smaller amount since the retracted skin should typically lie at more than 3-5 cm distance from the applicator.

With balloon applicators using either X-rays or Ir-192 the dose distribution can be improved by loading multiple source positions and generating a computer-optimized treatment plan. Dose distribution can be evaluated with dose-volume histogram-based dose metrics. With radioactive sources, multi-channel (multi-strut) applicators can be used to treat asymmetrical targets. A study comparing Ir-192 dose distributions for single lumen and multi-strut balloons with different dose calculation algorithms shows that dose coverage of a spherical shell of target depends more on the choice of optimization algorithm than on the number of source channels. Using inverse optimization with simulated annealing (IPSA), a D_90_ metric (dose to 90% of the target volume) of 99.95% vs. 102.56% of prescription dose is achieved for multi-strut vs. single lumen applicators [45]. With a simpler dose point based optimization D_90_ metrics of 88.4% vs. 90.27% are achieved. With the Xoft Axxent source and a single lumen applicator similar dose coverage (D_90_ = 101.2-103.9%) is calculated when assuming a homogenous water phantom [46]. For the low energy X-rays of the Xoft Axxent system this study calculates a reduction of D_90_ = 103.9% in homogeneous water vs. D_90_ = 98.9% calculated in a more realistic heterogeneous phantom. As a desirable effect, the heterogeneous calculations show a larger reduction D_0.2cc_ (maximum dose to 0.2cm^3^ of tissue) for skin dose from 92.9% (water) to 81.4% (heterogeneous) due to missing backscatter. A similar reduction should apply with Ir-192 where Skowronek et al. [45] find skin doses (D_max_) of 81.34-85.83% of prescription dose when calculated in water. This is confirmed by measurements of skin dose by Sadeghi et al. [47] who find average skin doses of 78.5% (range 56-488cGy, average 267cGy for a prescription dose of 340cGy) depending on tumor depth and size of applicator.

## Radiation protection

### Electron accelerators

An essential consideration in selecting and planning an IORT facility is the required radiation protection. Manufacturers of mobile IORT accelerators claim that their devices can be operated in an unshielded OR for limited numbers of around 3-5 patients per week. In reality, most ORs need some additional radiation protection. At minimum, safety devices like warning signals and transparencies, door interlocks and emergency stops are required. The manufacturers usually provide diagrams of stray radiation which can also be found in publications (Novac 7: [48], Liac: [4, 49], Mobetron: [50, 51]). Typical stray doses at 1m distance from the patient are similar for the commercial machines (since they are mainly caused by the bremsstrahlung produced in the patient (or phantom) as can be seen in the diagrams in [4] and [51]) and around 6μSv per Gy of patient dose [4, 51]. It is often difficult to pre-calculate the radiation levels in surrounding areas due to missing information on wall construction and the large range of possible machine positions and beam directions. Since the commercial machines are mobile, it is a reasonable consideration to ask the provider to perform test measurements with a test machine in advance of purchase and final calculation of shielding requirements. Regulatory bodies are usually prepared to grant test licenses which allow irradiation under additional safety precautions such as testing at times of low occupancy and blocking admission to surrounding areas. When preparing a radiation plan one must consider that the highest levels of stray radiation usually occur in the beam direction, immediately adjacent to the beam stop. Therefore, critical areas are often the rooms below the IORT OR. Radiation safety regulations usually require shielding which reduces ambient dose below the permitted levels at any occupied position around the OR under the assumption that the highest possible rate of stray radiation occurs in this direction during the complete workload. Radiation protection regulations generally allow use factors with which the workload in a certain direction can be reduced according to the fraction of time in which treatment beam is directed towards a particular barrier (e.g. NCRP 49 [52] below), however the constraints are different in different countries. E.g., German standards (DIN 6847-2 [53]) allow only a factor of either 1.0 or of 0.1, when use is less than 10% of the workload. In many situations, due to the variability of machine position and beam angle, agreement on the use factor is difficult to achieve between user and licensing authority, leading to the requirement of additional structural shielding in directions which in effect see only a fraction of the workload. The need for structural shielding could in principle be reduced by the use of mobile shielding walls as they are suggested by some manufacturers. Substituting structral by mobile shielding is frequently not permitted by licensing authorities due to the lack of control of correct placement when this is totally subject to the responsibility of the personnel. A possibility may be the development of interlocked systems which only permit irradiation if the crucial directions around the accelerator are protected by correctly positioned mobile shields.

The workload in the radiation plan must include sufficient dose to perform irradiations for quality assurance (QA), maintenance and repairs. An alternative can be a separate shielded vault where these tasks are performed. However such an installation will itself cause extra costs for space and radiation protection. Frequent transportation of the accelerator consumes time and manpower and typically will require shutting down the machine after QA and re-starting it in the OR which may change its operating conditions. More considerations for radiation protection are discussed in the reports of AAPM TG72 and the Italian Guideline [2, 3].

In radiation surveys it must be recognized that the IORT accelerators produce pulsed radiation. Some survey meters show incorrect readings in pulsed radiation in which the instantaneous dose rate in the pulse can exceed the instrument’s linear range [54]. Especially for measurements inside the treatment room, measurements of ambient radiation should preferably be performed with pulse-insensitive devices such as TLDs or with ionization chambers, which however can show the recombination losses discussed in the section on dosimetry.

### X-ray devices

A key reason for the use of kV X-rays in IORT is their lower requirement of radiation protection. As a first guess one would assume that a kV-IORT device needs roughly the same amount of radiation protection as a C-arm fluoroscopy unit as often used in the OR. Schneider et al. [55] confirm this assumption by measuring the dose rate surrounding the Intrabeam system and a C-arm unit in phantom arrangements simulating kyphoplastic radiotherapy, an intraoperative a breast treatment and a fluoroscopic procedure. For the C-arm they find dose rates of 12.7 mSv/h at the phantom surface and 56 μSv/h at 2m distance from the phantom. For Kypho-IORT the dose rates are 63.6 mSv/h (phantom surface) and 66μSv/h (2m), and for breast IORT 28.2 mSv/h (phantom surface) and 27μSv/h (2m). They estimate a workload of 260 treatments per year (1 patient per day, 5 days per week, 52 weeks) with 6 minutes per treatment for Kypho-IORT, 30 minutes per treatment for breast and in comparison 15 minutes C-arm use per day. With these numbers they compare the radius of the area around the patient/irradiator which receives more than 6 mSv per year and therefore defines a controlled area. They find a radius of 5.8 m for the C-arm, 6.7 m for Kypho-IORT and 3.4 m for breast IORT. For the breast simulations the phantom surface was covered by lead-rubber shielding equivalent to 0.175 mm of Pb which the authors state should reduce dose transmission to < 0.5%. Without such shielding, the controlled area and also the need for other shielding measures would be larger. Eaton et al. [56] report an ambient dose equivalent rate of 10.3 mSvh^-1^ at 1 m distance from the 3 cm applicator of an Intrabeam system placed on polystyrene blocks (to reduce scatter). With this set-up and with both a collimated and an attenuated beam they measure transmission factors for several shielding materials (tungsten-rubber sheets, lead, plasterboard, dry brick). They find that reference transmission factors for an X-rax unit at 50 kVp with 2mm Al filtration [57] are applicable (within a factor 2) for the treatment situation when the applicator is covered by patient and a sheet of tungsten rubber. The reference factors underestimate the transmission for an unattenuated and collimated beam. The report also includes measurements around a phantom set-up in which the applicator was either unshielded or inserted into wax bolus and covered by a sheet of tungsten rubber and also an environmental survey during 40 patient treatments. From the results the authors conclude that involved personnel (system operator and anesthesiologist) may stay in the treatment room with appropriate protection, other personnel must leave the OR. This decision is mainly to facilitate administration and to minimize training needs. As radiation protection in the OR, the institution uses mobile lead screens although also a lead apron would provide sufficient shielding. The patient treatment survey shows that due to varying positions of patient, treatment unit, lead screens etc., instantaneous dose rates can occasionally be higher than found in an initial survey. The authors conclude that for this reason controlled and supervised areas must be declared in a conservative way. One must expect that also for kV X-ray devices the licensing authorities will often be reluctant to permitting mobile lead shields and allowing personnel to remain in the OR during treatment. However one can argue that - in contrast to the use of a C-arm fluoroscopy unit in the OR – IORT is an extremely extraordinary situation during a limited and well-announced time period involving highly trained personnel under surveillance or even in presence of a medical physicist. This may justify that personnel can remain in the OR during treatment if sufficient protection is provided, however agreement with licensing authorities must be achieved in every individual case. In any case, also kV IORT devices require safety installations like warning signals and transparencies, emergency stops, and if possible, door interlocks.

## Commissioning, dosimetry, quality assurance

### Commissioning mobile electron accelerators

After acceptance testing, commissioning of an IORT device requires the measurement of all machine specific data needed for future treatment. For mobile electron accelerators, the report of AAPM TG 72 [2] gives a list of Items to be measured during commissioning including depth dose distributions and beam profiles at several depths, output factors for every used beam energy and applicator, leakage profiles at various depths outside selected applicators, air gap factors, and beam output calibration for every energy. These measurements require high doses and a large amount of time for which both staff and a measuring vault with sufficient radiation protection must be available and planned in advance. For the commissioning of an accelerator with 6 energies and 16 applicators, Hensley reports a dose consumption of 5145 Gy [58]. For modern mobile accelerators with around 40 applicators and 3 to 4 energies this would extrapolate to 6000-7000 Gy. A considerable reduction of time and dose could be achieved if the use of an atlas of pre-determined dose distributions were permitted which would only need to be verified for a smaller set of applicators. Such an atlas could be provided by the manufacturers or a task group authorized by medical physics societies similar to the practice in brachytherapy. It would require updates for machine changes and the obligation that manufacturers report relevant changes. Measured data could be cross-checked with Monte Carlo simulations. Such simulations exist for several mobile IORT accelerators for various purposes [10, 59–61] and could be further developed to predict and verify measured data sets. Commissioning especially of small and beveled applicators and measurement of output factors should preferentially be performed with small detectors which integrate only a small portion of the non-flat dose profiles. Electron diodes can be used after test of their performance in the pulsed beams. They should be frequently re-calibrated since their sensitivity may change with the high doses applied with IORT accelerators. Commissioning of beveled applicators is difficult and must be performed with great care since the correct position of the clinical axis (the intersection of the inclined central beam axis and the patient surface) is not easily found with the usual measuring tools. Correct position of the clinical axis (or some other reproducible point under the applicator) is essential in order to determine reproducible output factors for beveled cones. The mobile accelerators have no positioning aids such as light fields or alignment lasers. Beam centering tools to find the central beam axis provided by water phantom software cannot be used due to the asymmetric dose profiles. A positioning tool described by Runz et al. [62] uses templates mounted on the chamber drive of a water phantom to align the applicator end with the water surface and define the position of the detector in relation to the central beam axis which at the water surface coincides with the clinical axis.

### Dosimetry of IORT systems

#### Electron dosimetry

Reference dosimetry for electrons is performed following the international dosimetry protocol IAEA TRS 398 [63], or national protocols such as AAPM TG 51 [64] in the United States or DIN 6800-2 [65] in Germany. These protocols recommend calibration of electron beams under standardized conditions with either plane- parallel ionization chambers (or, for higher energies than usually provided with mobile accelerators with thimble chambers). Calibration is recommended for a standard rectangular field of 10×10cm^2^ (or for higher energies at 20×20cm^2^) which is not available with the mobile accelerators. The protocols provide energy correction factors k_Q_ for the ionization chamber at a reference depth z_ref_ which is determined from the 50% depth R_50_ on the central axis of the standard field. The depth R_50_ is used as parameter to determine the mean electron energy of the beam at the phantom surface. This energy determination is valid only under reference conditions and for broad beam geometry i.e. for fields large enough that depth dose no longer varies with field size. As shown in the report IAEA TRS 381 [66] the water/air ratios of electron stopping powers at z_ref_ which determine the beam quality correction factor (TG51: k_Q_ , TRS398: k_Q,0_ , DIN 6800-2: k_E,R_ ) depend on the beam energy but do not strongly depend on field size. Calibration of IORT accelerators should therefore be performed for the largest applicator and constancy of the depth dose distributions at this field size should be confirmed. Although the closed-wall applicators used in IORT will probably produce more scattered radiation than the applicators on conventional linacs and possibly do not completely provide broad beam geometry, one can assume that that the errors in the beam quality correction factor for the large applicators will be small. For beams of the Novac7 and the Liac with energies between 5 MeV and 10 MeV, Righi et al. [60] show that at z_ref_ the differences between Monte Carlo calculated (water/air) stopping power ratios and the values given in IAEA TRS 398 [63] are smaller than 0.4%, but increase at larger depths.

#### Dosimetry of pulsed electron beams

All the mobile linear IORT accelerators on the market produce high dose rates (around 10 Gy/min) at low pulse repetition frequencies between 9 and 40Hz and therefore produce large doses per pulse ranging between 3 and 60 mGy. For large pulse doses IAEA TRS 398 [63] and AAPM TG 51 [64] recommend correction for recombination losses of ionization chambers by the Two Voltage Analysis (TVA) method based on Boag’s theory [67–70]. This method overestimates recombination losses due to negligence of the free electrons in the gas filling of an ionization chamber (IC). For a Novac 7 accelerator with pulse doses between 30 and 60 mGy/pulse, Piermattei et al. report an overestimation of the correction factor k_S_ with the TVA method by of up to 20% [71] and suggest the determination of k_S_ by comparison of dose measurements with ionization chambers and calibrated radiochromic films. Improved calculation models for k_S_ were published by Di Martino et al. [72] and Laitano et al. [73].

Cella et al. [74] compare the predictions of the Di Matino model and three alternative models suggested by Laitano by comparison of measurements with two plane-parallel ionization chambers (Markus and Advanced Markus, PTW Freiburg) with a second set of measurements with pulse-independent ferrous sulfate (Fricke) dosimeters in beams of a Novac 7 accelerator (for pulse doses between 21.4 and 69.8mGy, measured with Fricke dosimeters). They find that the validity of the recombination corrections calculated by the different models depends on chamber type and factors such as chamber voltage.

Ghorbanpour et al. [75] compare the recombination correction k_S_ for a plane-parallel ionization chamber (Advanced Markus chamber, electrode spacing 1mm, PTW Freiburg, Germany) as calculated by TVA and by 3 alternative models from Laitano. They obtain k_S_ with an uncertainty of 2.1% by comparing dose measurements with calibrated radiochromic film with those of an uncorrected ionization chamber. For the pulsed beam of a Novac 7 (2.8 – 43mGy/pulse) they find deviations <3% for all three Laitano models, which agree among each other within 1%. For the TVA method they find larger deviations up to 4.2%. A comparison with k_S_ values determined with a Jaffe plot (described in IAEA TRS 398 [63], AAPM TG 51 [64], and DIN 6800-2 [65]) for pulse doses of 27 mGy shows a difference of 0.3%. From this finding, the authors conclude that Jaffe plots may be an appropriate method for determination of k_S_, but need further investigation for higher pulse doses. However, all findings must be seen in relation with the uncertainty of 2.1% in the author’s own determination of k_s_.

Bruggmoser et al. [76] modify the correction model by Burns and McEwan [77] and determine general model parameters (γ for the lack of charge collection and initial recombination and a recombination parameter δ) for 8 ionization chambers (4 plane parallel, 4 cylindrical chambers) from the analysis of chambers irradiated in the pulsed photon and electron beams of conventional medical electron accelerators. The modified Burns and McEwan model with these parameters together with chamber voltage and dose to water per pulse can be used to calculate the recombination correction k_S_. Calculations of k_S_ with this method are found to be in good agreement with published values for conventional beams. The calculation was tested for pulse doses of 41.3 mGy and 41.7 mGy in the beam of a Novac 7 for a Roos-type plane-parallel and a Farmer chamber (both PTW Freiburg) by comparison with pulse-independent thermoluminescence dosemeter (TLD) measurements. The corrected IC measurements agreed with the TLD measurements within 0.9% for the Roos and 0.3% for the Farmer chamber. This correction method is recommended for the 8 modelled detectors by the German dosimetry protocol DIN 6800-2 [65].

In summary the publications give no clear answer concerning the superiority of any of the models. The use of ionization chambers with small electrode separations and a high bias voltage should keep the recombination losses small but this must be confirmed for every installation.

Since the dose per pulse changes with depth in phantom, the measurement of relative dose distributions such as depth dose curves and profiles with an ionization chamber requires depth dependent recombination corrections. Therefore, measurement with selected diodes which must be checked for pulse dose and energy independence, are preferable.

Due to the uncertainties in determining the beam quality and recombination corrections it is recommendable to confirm the calibration of IORT accelerators by independent mailed electron dosimetry from an accredited calibration laboratory. Several calibration laboratories provide mailed dosimetry (e.g. TLD dosimetry service is provided by the IROC Houston Quality Assurance Center [78], Fricke dosimetry service is provided by the Italian INMRI [79], Alanine dosimetry by the British NPL [80]). Investigations to confirm the independence of the mailed dosimeters to pulsed radiation, and possibly the development of additional services would be helpful. The correction factors found by comparison with mailed dosimetry must then be adapted to all field sizes.

#### QA of electron IORT systems

Since IORT is applied in a single fraction of high dose, the output of IORT systems must be verified on a daily or even individual patient basis. Frequency of checks should only be reduced after careful verification of sufficient stability of the output. To fit into the busy time schedule of an OR, and to limit dose workload to permitted levels the dosimetric QA checks should be performed in quick and easily reproducible procedures. A check phantom which attaches to the end of a reference applicator is described by Hensley [58]. Output can be verified with an ionization chamber in one single depth for all beam energies in order to avoid changing the set-up. To additionally verify the beam energy, a measurement can be performed in two predefined depths. However, such a check requires repeated changes of chamber depth. A phantom allowing measurement at depth of maximum and around 50% on the PDD curve together with a dedicated QA applicator is supplied along with the Mobetron (shown by Beddar [81]).

With the high electron dose rates from IORT accelerators, charge accumulation in insulating (plastic) materials can alter the current measured by an ionization chamber [82–85]. Phantoms should therefore be constructed from conducting material or composed as stacks of slabs which locally accumulate only a negligible amount of charge.

For the Mobetron, Beddar [81] reports output variations within ±2% for all 4 beam energies in a sequence of 20 daily QA trials and a long term shift of calibration between 1.1% and 2.3% over a period of three years. To assess beam energy variations, he estimates the shifts in the depth dose curves leading to measured variations of the ratio D_max_/D_50%_ measured with the dual depth phantom mentioned above and finds shifts well within ±1mm. Other accelerators seem to be less stable in output and possibly also in beam energy. Agostinelli et al. [86] show a variation of daily output of ±5% for the Liac. They find a correlation these variations to a control parameter (COR, a measure proportional to the difference between preset and measured dose per pulse) displayed on the control console. Possible causes for the unstable output that are in discussion, but have not been described in literature so far could be recombination losses in the monitor chambers which lie immediately behind the beam exit window and are therefore exposed to extremely high dose pulses. This may be increased by the low pulse repetition rate (10 Hz for 10 MeV beam, 35 Hz for the 8 Mev beam) which leads to a low rate of correction cycles in the dose rate stabilization possibly leading to large changes in pulse dose in repeated corrections which do not average out. The manufacturer states to have improved the monitor system, however no reports have been published so far. Another possible cause of instability may be changes in beam energy visible in measurements outside the depth of D_max_. These could be caused by the flexible wave guide connecting the magnetron and the accelerator guide. With this construction the wave guide will bend differently in different head positions and thereby change its resistance which can possibly lead to energy shifts. However, no reports concerning these hypotheses were found in literature.

### kV X-ray systems

The kV X-ray systems come pre-calibrated by the manufacturers with pre-commissioned sets of dosimetric data. Parts of dosimetric commissioning are identical with dosimetric QA checks. Additional checks are described separately for each system.

### Intrabeam

#### Commissioning

For the Intrabeam system an atlas of depth dose distributions for every applicator is integrated in the control software. Verification of dose distributions at distances of a few centimeters or less from the radiation source requires extremely precise positioning of source and detector within less than 0.1mm. Arrangements with this precision are difficult to implement in standard water phantoms. Zeiss therefore supplies a special water phantom with a source support which can position and move the source horizontally and vertically with the required precision and rotate it continuously by 360° with 8 precision positions in 45° steps. All movements are defined and indicated in relation to two fixed chamber positions (lateral and longitudinal) for a soft X-ray ionization chamber (in older systems for a 0.2cm³ PTW 23342, the same as used in the PAICH QA tool of the Intrabeam system. Newer phantoms are provided with a 0.005cm³ chamber PTW 34012, Both chambers are manufactured by PTW, Freiburg, Germany). Movements are possible with or without applicator. The IC is positioned in the phantom inside two horizontally arranged waterproof (Perspex) chamber holders with a thin solid water entrance window facing the source. The IC is delivered along with every system.

#### Dosimetry and QA

Recommendations for the dosimetry of low energy X-rays are given in IAEA TRS 398 [63], AAPM TG report 61 [87], the code of practice published by the IPMB [88] and the German Standard DIN 6809-4 [89]. These recommendations were primarily written for the dosimetry of superficial (skin) therapy, and therefore describe dosimetry at the surface of a phantom. The reference dosimetry of all three kV IORT systems (Intrabeam, Papillon, Xoft) provided by the manufacturers or published independently deviates from these recommendations. Verification by users is not straightforward and requires careful consideration of the energy dependence of detectors in the kV region, the choice of phantom materials and the application of often unpublished corrections. Eaton [19] gives a comprehensive and detailed description of dosimetry and QA for the Intrabeam system which can be used as reference for further information.

#### Zeiss calibration of the Intrabeam system

Zeiss provides the Intrabeam system with a calibration using essentially the setup published for the older PRS system by Beatty et al. [17, 90]. Dose is measured in water without applicator with a soft X-ray chamber (PTW 23342, the same type as provided by Zeiss with the Intrabeam) at depths of 3-45mm in steps of 0.5mm. Measurements are performed in a water phantom (similar to the one provided to users by Zeiss) in which the chamber is contained in an acrylic water protection with a thin solid water beam entrance window at a fixed position, and the source is moved with a precision drive. The chamber is calibrated in air in terms of exposure, its reading is converted to absorbed dose to water with a conversion factor *f* = 0.881 mGy/R. For the half-value layers (HVLs) between 0.1 an3 mm Al measured for the Intrabeam (see Table 1 and section *HVL Measurements*) the mass energy absorption ratios ${\left({\overline{\mu}}_{en}/\rho \right)}_{water}/{\left({\overline{\mu}}_{en}/\rho \right)}_{air}$ governing the variation of *f* vary within around 2.6% [87]; the beam quality correction specified by PTW is 1.000 in the region T30 to T50 (HVL 0.42-0.93 mm Al). No corrections are documented by Zeiss for polarity or the attenuation of the beam entrance window. The dosimetry protocols recommend the calibration of low energy X-ray beams at the surface of a semi-infinite water equivalent phantom (providing full backscatter), AAPM and IPEMB additionally recommend a correction for perturbation of the radiation field by the chamber and the changes of chamber sensitivity caused by the scatter radiation from the phantom (named *P*_*Q*,cham_ in AAPM TG 61 [87]and *k*_*ch*_ in the IPEMB code [88]). Values of *k*_*ch*_ recommended by IPEMB [91] for the PTW 23342 chamber and a semi-infinite phantom range between 1.01 and 1.07 for HVLs of 0.1mm Al and 1mm AL. Eaton and Duck [43] measure values of around 1.05 for the Intrabeam with 1.5cm and 5.0cm spherical applicators at 3.8cm distance from the surface of a solid water phantom. Zeiss does not document the use of such a factor which in this case would be caused by the water phantom and the acrylic holder surrounding the complete chamber (notably the chamber has no guard ring to exclude scatter). The depth dose curve measured in the water phantom without applicator is fitted by an analytical function in order to extrapolate the dose down to the probe surface. The dose measured with the calibration described above (at a depth not named by Zeiss) is related to a second measurement with the user’s PTW 23342 ionization chamber in the PAICH QA tool described in the section quality assurance. This allows output control in daily QA. To determine the dose with an applicator, a second depth dose curve with applicator is measured with the same procedure as above with a quality controlled standard Intrabeam source owned by the manufacture. The ratio of the two depth dose curves is fitted to a second analytical function called the applicator transfer function. Multiplication of the applicator transfer function with the user’s depth dose curve generates the depth dose curve with applicator at the user site and via PAICH measurement relates the output to the dose. With this method the minimum uncertainty is stated as 4.2% for a dose measurement at precisely 2cm depth in water and 5.3% at other depths due to positioning uncertainty and change of chamber response with depth. In this way Zeiss provides a reproducible system with reliable quality control in which applicators and X-ray sources can easily be interchanged. A drawback is that it is difficult to relate an independent dose measurement to the daily QA measurement with the PAICH. To verify the output and depth dose curves, Zeiss provides correction factors with which the reading of a smaller air-Kerma calibrated PTW 34103 ionization chamber provided together with the water phantom is multiplied. The factor converts the measured “non-Targit dose” to “Targit dose” as it is applied in the Targit breast treatments [92]. The correction factors are depth dependent and vary between 0.5 at 3mm (0.77. at 7.5mm) and 0.9 at 45 mm distance from the source. Their uncertainty is stated as around 6.7%. Zeiss explains that the factors correct for a combination of three effects. 1: a different effective point of measurement in the two ionization chambers caused by the different solid angles of beam seen by the 34013 and the 23342 chamber due to their entrance foils of 2.9 vs. 5.2mm diameter (leading also to the distance dependence). 2: Different designs of the chamber holder producing different measured dose rates. Zeiss states that this is particularly noticeable with small source distances. 3: the different calibrations of the 23342 chamber in terms of exposure and the 34103 chamber in air Kerma. Zeiss states that the different conversions of exposure or air Kerma to absorbed dose to water by factors of *f* = 0.881 mGy/R (23324) or k_Ka→Dw_ =1.036 (34103) leads to a constant offset in the entire measuring range. From the Zeiss statements it is not clear which of the measured quantities corresponds to physically correct absorbed dose to water (or if either of them does). The explanation conflates different causes for the correction factors and no publications are named for their confirmation. Therefore, a confirmation of the Intrabeam dosimetry system by independent methods is highly desirable. Supported by Zeiss, the German standards laboratory PTB (Physikalisch-Technische Bundesanstalt, Braunschweig, Germany) is developing a primary standard for kV dosimetry which should provide the correct values of the doses delivered by the Intrabeam system [93].

**Table 1:** Half value layers (HVL) in Al and mean or effective energies for the spectra of IORT devices using KV X-rays

Publication	System	Probe	Surrounding material	Distance from scource	Acceleration Voltage	HVL (mm Al)	Mean or effective photon energy	Other remarks
Zeiss Dosimetry manual [90]	Intrabeam	bare probe	water	10 mm fom tip	50 kV	0.64 mm	20.4 keV	beam quality T30 -T50 (PTW definition)
					40 kV	0.48 mm	19.1 keV	≈T30
					30 kV	0.41 mm	17.3 keV	≈T30
Beatty 1996 [17]		bare probe	air		40 keV	0.1 mm		
				2nd HVL	40 keV	0.16 mm		
Armoogum 2007 [18]		bare probe	air					
Beatty 1996 [17]		bare probe	in air behind 0.5 cm solid water		40 kV	0.3 mm	14.8 keV	
			in air behind 1 cm solid water		40 kV	0.71 mm	19.9 keV	
			1.5cm solid water				21.7 keV	
			2cm solid water				23.5 keV	
Armoogum 2007 [18]		bare probe	in solid water	1cm		1.11 mm	23.5 keV	broad beam
				5mm		0.54 mm	18 keV	
				in air		0.11 mm	10.75 keV	
Avanzo 2012 [95]		bare probe	in solid water	1cm	50 kV	1.75 mm		
				2cm		2.14 mm		
						3.82 mm		
Ebert 2009 [101]		bare probe	in water	0 cm	50 kV		19.5 keV	
				5 mm			27.3 keV	For other depths, changes in spectra were calculated using linear attenuation in water and inverse-square of distance
				15 mm			31.5 keV	
				30 mm			34 keV	"
Avanzo 2012 [95]		bare probe	air		50 kV	0.11 mm	10.2 keV	
		3.5 cm appl.	solid water			0.8 mm	20.7 keV	
		4 cm appl.				0.98 mm	22.5 keV	
		4.5 cm appl.				1,1 mm	23.7 keV	
		5 cm appl.				1,23 mm	25.1 keV	
		bare probe	1 cm depth in solid water			1,75 mm	28.0 keV	
		bare probe	2 cm depth in solid water			2,14 mm	29.3 keV	
		bare probe	beind Tungsten rubber shield			3,82 mm	36.3 keV	
Eaton 2010 [43]		1.5 cm appl.	air		50 kV	1.1 mm		narrow beam
		3cm appl.	air			1.3 mm		narrow beam
		3.5 cm appl.	air			0.85 mm		narrow beam
		4.5 cm appl.	air			1.24 -1.25 mm		broad beam
		5cm appl.	air			1.26-1.29 mm		broad beam
Croce et al. 2012 [28]	Papillon				50 kV			
Carver et al. 2013 [98]		22/25 mm Appl.				0.64 mm		
Liu 2008 [111]	Xoft Axxent	bare source	air		40 kV	0.45 ± 0.07 mm	16.8 ± 0.9 keV	
					2nd HVL	0.7 ± 0.1		
					50 kV	0.5 ± 0.2 mm	18 ± 2 keV	
					2nd HVL	0.9 ± 0.3 mm		
			water	1.5cm	40 kV	1.04 ± 0.04 mm		

#### Independent dosimetry

A number of independent dose measurements in different set-ups have been published of which some show discrepancies with the Zeiss calibration.

Dosimetry protocols recommend the calibration of soft X-ray beams (10-100kV, half value layer HVL < 1-3mm Al) in air at the surface of solid or water phantoms, and not in depth in water due to the uncertainties in positioning and the uncertainties in attenuation and perturbation by the waterproofing of soft X-ray detectors [63, 87–89].

#### Publications on dose measurements

The first report by Beatty et al. [17] on reference dosimetry for the older type of the PRS system measures the dose (without applicator) at 10mm depth in water with an older type (PTW 30-334) of the soft X-ray chamber provided by Zeiss (PTW 23342). The chamber is calibrated in air in terms of exposure. As in the Zeiss calibration, its reading is converted to absorbed dose to water with a Roentgen-to gray conversion factor (*f*) without any of the corrections for differing beam quality (P_Q_), ion recombination (P_ion_), or polarity (P_pol_) recommended in the later dosimetry protocol AAPM TG 61 [87]. During the measurement the chamber is contained in a waterproof acrylic housing with a thin solid water window. No corrections are made for changes of the scatter radiation caused by the acrylic holder or the changes of amount or spectrum of the direct radiation through the window (which are partially included in the *P*_sheath_ correction in AAPM TG61 [87]). Correction factors for homogeneous PMMA, nylon and polystyrene sleeves surrounding a Farmer (thimble type) IC are reported by Ma and Seuntjens [94], however no reports were found for sleeves with a thin solid water entrance window. The authors estimate an overall uncertainty of ±10.2%, resulting mainly from the positioning uncertainty with a contribution ±2% from the IC calibration and ±1% from the conversion factor f.

Ebert and Carruthers [42] report a verification of IC measurements in water in the same set-up as the Zeiss calibration by simulations with an analytical beam model and Monte Carlo calculations. They show that due to spectral changes, the ratio of mean mass-energy absorption factors of water to air (which determines the energy dependent response of an ionization chamber) decreases by about 2.2% between the surface of the bare source and 3-4cm depth in water. Monte Carlo calculated depth dose curves with applicator in comparison to curves measured by the manufacturer show good agreement, however there is discrepancy in the change of dose rate altered by larger applicators in relation to the dose rate of the bare probe.

Eaton and Duck [43] describe a method of calibrating the system using the IPEMB protocol by calibrating an ionization chamber and radiochromic films (EBT) in air with a second X-ray device with similar HVL (see section *HVL measurements* below), including a determination of chamber correction factors *k*_ch_. They use these calibrations for reference dose measurements at the surface of a solid phantom (Solid Water WT1) with the IC. Relative dose distributions in water are measured with film and IC in a water phantom. The paper states no value for the uncertainty of reference dosimetry, however differences within 6.9% between IC and film measurements. The differences between film and manufacturer data are up to 4.8% at distances larger than 10mm from the applicator surface and up to 8.8% at distances less than 10mm. It is not clear if these differences relate to the absolute dose values stated by the manufacturer or the normalization at 10mm distance from the applicator surface. The authors attribute the differences mainly to uncertainty in positioning in the steep dose gradient but the difference in film response between the calibration in air and the measurement in the hardened spectrum in water should be considered as a possible cause.

#### Dose verifications with in-vivo dosimetry

Two publications on in-vivo dosimetry attempt to verify the dose delivered by the Intrabeam system and find large deviations:

With EBT2 film, Avanzo et a. [95] report in-vivo measurements in 23 patients at the surface of Intrabeam applicators which differ from the doses specified by the Intrabeam software on average by -27.6%, -19.9%, -11.9% and -10.4% for the 3.5cm, 4.0cm, 4.5cm and 5.0cm applicators. The films are calibrated with the 3.5cm Intrabeam applicator (at 50kV_p_ acceleration voltage) in air by comparison with measurements with a PTW 23324 ionization chamber. The authors assume that incomplete adherence of film and applicator or incorrect positioning of the detector may be the cause for the deviations. It is not reported if corrections were made for dose averaging on a flat film irradiated in the steep and divergent gradient around the spherical applicator which may have caused a reduced signal. Similar deviations are found by Price et al. [96] with measurements in 20 patients at the applicator surface with RTQA2 films and with OSLDs (optically stimulated light emitting dosimeters). Dose differences found with films were +6.3%, -7.3%, -11%, -16.6% at the surface of the 3.0cm, 3.5cm, 4.0cm and 4.5cm applicator, with OSLDs: -1%, -21% -30% and-13.6% at the surface of the 3cm, 4cm, 4.5cm and 5cm applicator. Both films and OSLDs are calibrated in water in the beam of the 4cm applicator (at 50 kV_p_) by comparison with measurements with a PTW 34013 chamber. It is not documented if the readings of the chamber were corrected with the Zeiss factors (or if these factors were available at the time of publication in 2010). Again it is not documented if dose averaging effects on the film were corrected. Although in the results of both publications a number of uncertainties must be considered (adherence of the detector to the applicator surface, energy dependence of the detector, volume averaging, tissue displacement by the OSLDs), also calibration uncertainties of the Intrabeam system should also be considered as possible cause of the differences.

#### Summary: Intrabeam dosimetry

A number of uncertainties remain in the reference dosimetry of the Intrabeam system which require clarification. Publications of reference dosimetry are needed with an applicator in water using the ionization chamber and water shielding sheath provided by Zeiss and also a check device to quickly verify the PAICH dosimetry would be helpful. A Perspex phantom which is attached to the Intrabeam probe could possibly be calibrated for this purpose is described by Armoogum and Watson [97]. It must be stressed that the uncertainties do no not preclude therapeutic use of the system, since the dosimetry system currently recommended by Zeiss applied by the users is empirically proven as safe and is efficient within the assumptions of the Targit and other trials. In case a correction of the “Targit dose” to a different physically defined absorbed dose is needed, this may not lead to a change of dose prescription until medically justified. However, a clear correlation of “Targit dose” to physical dose is important to understand dose-effect relationships and to compare Intrabeam treatments with other methods.

#### Dosimetry of the Papillon system

In general, the same considerations and methods should apply for dosimetry of both the Papillon and the Intrabeam system. For this system, Carver et al. [98] investigate the conditions for dosimetry with Monte Carlo calculations and calculate chamber corrections *k*_ch_ and backscatter factors. Croce et al. [28] compare Monte Carlo calculated dose distributions in PMMA and water with relative dosimetry using a plane parallel ionization chamber (PTW 23342, PTW Freiburg) and EBT2 radiochromic film in a PMMA phantom. The choice of PMMA as phantom material may be a source of uncertainty as shown by Schönfeld et al. [99]. Due to its higher density of 1.19g/cm^3^, dose distributions in PMMA can differ considerably from those in water, depending on distance from the source and also on phantom size.

#### General aspects of kV dosimetry

##### HVL measurements

Due to the energy dependence of the response of most detectors, care must be taken to calibrate in a similar radiation quality as the measured. The response of most dose detectors depends on photon energy, and the parameter recommended by the dosimetry protocols to describe the beam quality is its half value layer (HVL) in aluminium. For some detectors, manufacturer document energy dependent response in terms of the photon energy. As shown by Chofor et al. [100], for many detectors and for energies above 100 keV the mean energy of the fluence spectrum determines the energy response. Table 1 summarizes published measurements of Intrabeam HVL of and shows that there is a substantial variation depending on surrounding material, applicator and distance from the source. Zeiss [90] provides measured HVLs at 10mm depth in water (apparently without applicator) for the type 4 X-ray source (XRS) of the Intrabeam together with corresponding effective energies E_eff_ and beam qualities as used by PTW Freiburg for the calibration of the PTW 23342 0.2cm^3^ plane-parallel ionization chamber (IC) (PTW Freiburg, Germany) which is supplied along with the Intrabeam system. For an acceleration voltage (AV) of 50 kV, Zeiss states an HVL of 0.64 mm Al, corresponding to E_eff_ = 20.4 kV or a beam quality index between T30 and T50 (for AV = 40kV : HVL = 0.48mm Al, E_eff_ = 19.1 kV, ≈T30; for AV = 30 kV: HVL = 0.41 mm Al , E_eff_ = 17.3 kV, ≈T30) [90]. Although this is not explicitly indicated by Zeiss, these values probably apply to the bare probe surrounded by water. Measured half value layers (HVL) for the Intrabeam system have been reported by several authors [17, 18, 43, 95, 101] showing the massive spectral changes by beam hardening in the applicators and in tissue (or water) which are already shown in air in the report by Beatty et al. [17]. Table 1 compiles the half value layers and mean energies published by various authors and shows that the beam is substantially hardened by the applicator and by increasing depth in water.

For the unattenuated beam, Beatty et al. [17], Armoogum et al. [18] and Avanzo et al. [95] report HVLs of 0.1-0.11mm Al. With the source inside the breast applicators, Eaton and Duck [43] report 1.3 mm Al for the 3cm applicator, 0.85mm for the 3.5cm, 1.24-1.25mm for the 4.5cm and 1.26-1.29mm for the 5cm applicator. Avanzo et al. [95] report: 0.8mm (3cm appl.), 0.98mm (4cm appl.), 1.1mm (4.5cm appl.) and 1.23mm (5cm appl.). For the bare source behind 1cm solid water, Beatty et al. [17] report an HVL of 0.71mm Al, and Armoogum et al. [18] report 1.11mm Al. Anvanzo et al. [95] report 1.75mm Al at 1cm depth in solid water, 2.14 mm at 2 cm depth in solid water and 3.82mm behind a tungsten shield for a source in an applicator. The compilation in Table 1 shows that HVL and mean energy are different at practically any two measuring positions. This causes the need of dosimetry detectors with response independent of photon energy in the region of 1-50 keV and makes the comparison of different methods of calibration of the system difficult.

##### Dosimetry with radiocromic film

A number of the publications on calibration, measurement of dose distributions and in-vivo dosimetry for kV-X-ray devices report the use of radiochromic film, many using types of film which are known to have a strongly energy dependent response in the low energies occurring with these devices [95, 101, 102]. Recent publications show that the response of EBT film in comparison to a calibration in Co-60 increases from -39% at 20 keV to around -18% at 40 keV [103], the first value corresponding to the mean X-ray energy at the surface of the bare Intrabeam source (in air). In the region 20 keV to 25 keV (corresponding to the effective energy at the surface of the Intrabeam applicators) the energy response would then vary between -40% and -35%. This variation of locally around 14% could have caused part of the differences between Zeiss specifications and measured depth dose curves found by Eaton and Duck. Ebert et al. [101] find a similar dependence of response of EBT films between 0 and 30mm distance from the Intrabeam probe in water and conclude that this film is not suitable to obtain quantitative dosimetry information at the low energies involved here. Avanzo et al. [95] show calibration curves of EBT2 film for 6MV photons and 50kV photons measured with the bare Intrabeam probe in air (HVL = 0.11 mm Al) in which the optical density differs by about 10%. A second set of measurements in the beams of 3.5cm and 5cm applicators showed no significant energy dependence in the corresponding energy range (3.5cm applicator: HVL 0.8mm Al, mean energy 10.2 keV; 5cm applicator: 1.23mm Al, 25.1 keV). A report on the energy dependence of EBT and XR-QA radiochromic films to kV in comparison to MV photons is given in the report by Chiu-Tsao et al. [104]. Uncertainty in the reading of radiochromic film is strongly influenced by the selection of scanner and the scanning procedure. General recommendations for the use of radiochromic film are given in the report of AAPM TG 55 [105] , detailed reports on film handling and reading of optical density for radiochromic films used in IORT are given by Avanzo et al, [95], Ebert et al. [101], Ciocca et al. [106], Price et al. [96], and Severgnini et al. [107]. It is important that the type of radiochromic film is reported in publications, and care should be taken to use films and other detectors with appropriate energy response. Overviews of different films used in IORT are given in the reports by Eaton [19] and Ebert et al. [101].

##### Thermoluminescence Dosimetry

Soares et al. compare dosimetry of the Intrabeam with thermoluminescense dosimeters (TLDs) and two ionization chambers in a water phantom. IC reference dosimetry is performed under thin layers of water equivalent plastic. They find reasonable agreement between the results with the two detectors and estimate an overall uncertainty of 8-13% for the TLD measurements, however make no comparison with the Zeiss dosimetry [108]. This publication and also Eaton et al. [109] report assessments of the energy dependence of the response of TLDs in the energy region of kV-IORT and find variations of around 29% [108] and 6-9% [109] for LiF:Mg,Ti and around 98% for LiF:Mg,Cu,P [108].

##### Phantom materials for low energy X-ray devices

As described by Eaton [19] in more detail, the correct selection of phantom material for dose measurements of low energy X-rays is crucial since dose deposition is dominated by photoelectric absorption and therefore very dependent on material composition. Additionally, the X-ray spectra can change differently with distance from the source in the phantom (or applicator) material and in water. An investigation by Schönfeld et al. [99] for Ir-192 shows that the depth dose distributions in many solid phantom materials considered as water equivalent in external beam therapy can considerably differ from water. The spectral changes for photons with energies > 100 keV are mainly caused by increased scatter from Compton interactions, so that the mean energy will decrease. For low energy X-rays the mean energy will increase by stronger photoelectric absorption of the lowest energy photons. This will probably lead to different depth dependent variations of dose deposition in material which may not be negligible and need further inspection. For Ir-192, suitable commercial materials requiring a dose correction of less than 1% for measurements up to distances of about 5-10cm in phantoms with dimensions smaller than 20cm radius are Plastic Water LR, Plastic Water DT (both CIRS, Norfolk, VA), RW1 and RW3 (both PTW, Freiburg, Germany). The same phantom materials are quoted by Eaton [19] for investigations with the Intrabeam system. Both authors find that Perspex should not be used as phantom material due to its higher density and backscatter. The same considerations as for phantoms hold for the material of the waterproofing sheath for an IC used in a water phantom.

##### Summary

In summary, measurements of both reference dose and dose distributions for kV X-ray systems are difficult and care must be taken in the selection of detectors and phantom materials with small energy dependence in the range of 10-50 keV or appropriate corrections. The photon spectrum changes for each applicator and additionally with distance from the source. Care must be taken to select a radiation quality for calibration for which detector sensitivity can be corrected to the measured quality. Further publications would be helpful both on detector calibration and on manufacturer independent dosimetry of the kV systems with applicators in water.

##### Quality assurance

The Intrabeam system is delivered with a calibration by the manufacturer and includes two tools for daily QA, the PDA (Photodiode Array) and the PAICH (Probe Adjustment and Ionization Chamber Holder). Both tools are self-shielding (the drift tube with the X-ray target is completely inserted into the tools) and allow QA in an unprotected environment with negligible exposure to the operator. The PDA contains 5 diodes arranged around the tip of the drift tube (4 in the horizontal plane, one in front) which measure the isotropy of output and can be used for electronic adjustment of beam deflection. If anisotropy larger than the pre-set tolerances is detected, the drift tube (probe) can be mechanically adjusted in the PAICH tool. Here the position of the source tip is inspected with a light source and photo diode and can be manually aligned with a plunger. The PAICH also has a shielded insert for a PTW 23342 soft X-ray chamber (supplied with the system together with a PTW Unidos E electrometer) with which the source output is measured and the internal radiation monitor (IRM) is calibrated. The IRM is a photodiode detector in the source housing which continuously measures source output. Temperature and air pressure corrections are performed automatically by the control system. Tests of isotropy and output can be performed in around 10 minutes and are required before every new patient together with a number of administrative and safety checks. The checks are controlled and recorded automatically by the system computer during patient set-up. Note that the QA tests verify only the output in air, to which the dose in water with or without applicator is so far only related by the manufacturer calibration.

Additional to the daily tests, Eaton [19] recommends a number of procedures at longer intervals including six-monthly reassessments of environmental dose and checks of chamber constancy, annual tests (or following system service or re-calibration) of beam steering, monitor linearity and measurements of source output in water or solid phantom and source isotropy and depth dose in water. Additionally, he recommends a biannual intercomparison of chamber calibration using a second superficial X-ray unit.

### Xoft Axxent system

#### Calibration

Reference dosimetry for the Xoft Axxent X-ray source is based on an air Kerma-rate standard at 50cm from the source provided by the national American dosimetry standard laboratory NIST [110]. DeWerd et al. propose a modified TG43 dose calculation protocol [32] in which the absorbed dose-rate to water at the TG43 reference point, ${\dot{D}}_i\Big(1 cm,\frac{\uppi}{2}$) , is calculated as the product of the air Kerma-rate at 50cm source distance ${\dot{K}}_{50}$ with an applicator-dependent dose-rate conversion coefficient ${\chi}_i={\dot{D}}_i\Big(1 cm,\frac{\uppi}{2}$)/${\dot{K}}_{50}$ wherein the index $i$ designates the applicator [111]. The University of Wisconsin Accredited Dosimetry Calibration Laboratory (UWADCL) will use these dose–rate conversion factors to provide calibrations of the well chamber (HDR-1000; Standard Imaging, Middleton, WI) which comes with every Xoft Axxent system. For the water-like balloon applicators used in breast IORT, the TG43 data published by Rivard et al. can be used [32]. These are measured and calculated for water surroundings without applicator and for source voltage settings of 40 kV, 45 kV and 50 kV. For some applicators (e.g. titanium cervix applicators) also provided by Xoft , applicator-specific radial dose functions g_i_(r) and anisotropy functions F_i_(r,θ) as defined in the modified TG43 dosimetry protocol must be used.

With the well chamber, the output of the individual source is measured before clinical use and before every treatment. With this source calibration and the TG43 data, treatments can be planned with a commercial planning system supporting TG43 calculations. The paper by Rivard et al. [32] also describes a calibration of the Xoft source in a precision positioning water phantom with a plane plate IC (PTW 34013, PTW Freiburg, Germany) housed in a solid water waterproofing. Calibration of the chamber is traceable to the German National Standard Laboratory PTB (Physikalisch-Technische Bundesanstalt, Braunschweig, Germany). For this reference dosimetry in water, Rivard et al. [32] state an uncertainty of 4.6%, the uncertainty of the dosimetric parameters is stated as 4.7% at 1cm and 8.2% at 5cm distance from the source. Together with an estimated uncertainty of 2% for the calibration of the well chamber, quadratic addition yields a total uncertainty of 6.6% at 1cm and 9.4% at 5cm distance. Table 1 shows the HVL and mean energy of the Xoft Axxent as reported by Liu et al. [112].

#### Quality assurance

The Xoft Axxent system is supplied with a physics and QA accessories kit which includes a phantom arrangement called test fixture consisting of 3 plates of acrylic phantom contained in a shielding which allows irradiation in the fixture with minimal exposure to the outside. The middle slab is 5.2mm thick and has a central drilling that can accommodate the source (diameter 3.2mm) covering the source by 1mm of acrylic. A grid of radiopaque position markers on the slab allows inspection of correct source end position and pullback visually or by exposing a radiochromic film. By placing a sheet of radiochromic film on the opposite top of the source guiding slab, exposure distributions can be recorded to verify e.g. source pullback or the isotropy of the dose. Caution should be taken in relating the film signal to dose distributions since, as discussed for the Intrabeam, energy response of radiochromic film is energy dependent and spectral changes in a PMMA phantom at different distances from the source (as shown by Schönfeld et al. for an Ir-192 brachytherapy source in water [99]) may cause additional changes in response. The bottom slab of the phantom contains an accommodation for an ionization chamber, placing it at a position below the center of the source drilling. An assignment of the chamber reading to absorbed dose to water is uncertain (and not mentioned in the manufacturer documentation) due to unclear absorption, spectral changes and scatter conditions pertaining to the low energy X-rays which may cause changes in the chamber response (as shown by Chofor for Ir-192 sources [100]). Additionally, tools are provided to ensure correct source positioning in an applicator by measuring the length of the individual source arrangement (source plus guiding structure), the length of the source channel in the applicator (“applicator depth”) and to calibrate the outdrive and step length of the source retraction (“pullback”) mechanism on the controller arm. Such tests and adjustments are needed before patient treatment for every new source and applicator. Output is measured in the well chamber before every treatment; the standard deviation of dose rate for a given source during a series of measurements was found by Rivard et al. [32] to be < 0.3% at all operating voltages.

### In-vivo dosimetry (IVD)

For both IORT modalities, electrons and kV-Xrays, a large number of examinations have been published dealing with in-vivo dosimetry, possibly indicating a need for better information in IORT dose and dose distribution. As detectors, MOSFETs [86, 113–118], radiochromic film [106, 107, 115–118, 119], optically stimulated luminescence detectors (OSLDs) [96] and TLDs [118, 120] have been used, in one publication the use of semi-conductor diodes is reported [121]. Additional file 1 gives a compilation of the publications showing the detectors and their uncertainties as stated by the authors, the investigated treatment sites and the measured quantity, and highlights the detected deviations from the expected dose. Some authors derive action levels for correction of the treatment setting which are also shown in the table. Details of the table items are discussed in the following sections, the deviations between expected and measured dose for kV irradiations are discussed in the section on dose verification by in-vivo dosimetry of the Intrabeam system.

#### Detector selection and uncertainty for in-vivo dosimetry

In addition to the requirements discussed in the sections on dosimetry i.e. small statistical uncertainties, a small energy dependence of response, especially in the kV X-ray region and the selection of a comparable radiation quality for calibration, detectors for in-vivo dosimetry in IORT must have a number of special properties connected to their measuring position inside the patient tissue. Criteria for detector selection in in-vivo dosimetry are small size (in order to place it inside the surgical cavity or even a catheter), its ability to be sterilized, and also a small temperature dependence, since calibrations are normally performed for reference conditions (20 °C) and measurements at body temperature (37 °C).

For MOSFETs, uncertainties in response between 1.5% and 5% are reported (all for electrons) [113–116, 122]. For these detectors, two publications report a dependence of response on the angle of radiation incidence of up to >15% at 45° and 24% at 90° which can also contribute to uncertainty [115, 122].

With radiochromic film, uncertainties of around 3% are reported for electrons [106, 117, 118]. For kV X-rays one would expect larger uncertainties due to the energy dependence of film and TLDs discussed in the section on kV dosimetry which depends on type of film . Avanzo et al. report a reproducibility of 2.8% of the reading of EBT2 films calibrated in air at 30mm distance from the bare probe of an Intrabeam irradiator [95, 97].

The energy dependence of the response of TLDs in the energy region of kV-IORT is reported in three publications [108, 109, 123] and discussed in the respective section on general dosimetry of kV X-ray systems.

Price et al. [96] investigate the energy dependence of OSLDs and RTQA2 radiochromic film in the strongly varying spectra of kV X-ray units by measuring at several fixed distances from two Intrabeam applicators of different diameter. They find changes in response of OSLDs between -6.6% and 2.8% at 0.15mm distance from the applicator surface and between -16.5% and 3% at 1cm and 2cm distance. For RTQA2 radiochromic film they find differences in responde between -6.8% and 6.2% at the surface and -1.8% and 8% at larger depths.

A source of uncertainty may be the distortion of the radiation field by the detector. Consorti et al. [115] report an increased attenuation of up to 20% for 4 MeV electrons and around 1.5% for higher energies caused by a MOSFET detector contained in a plastic catheter. For RTQA2 films in a solid water phantom, Price et al. find dose reduction of 8.5% at 5mm distance from the needle tip decreasing with distance to 2.6% at 2cm distance [96]. For OSLD detectors they find an increase in reading between 4.1 and 20.5% caused by fact that the rather large housing of the detector has a lower density than the water it displaces. For in-tissue measurements they suggest to consider only the increased absorption of 5.9% by the detector itself since the housing mainly displaces tissue and due to its reduced absorption causes a smaller change to the surrounding local dose.

All mechanisms listed above may contribute to the uncertainty and the dose deviation found in in-vivo dosimetry.

#### Action levels

Several of the publications on in-vivo dosimetry of IORT with electrons report entrance dose measurements some of which are apparently aimed at correcting for the unstable output of some machines [86, 106, 113, 114]. For such measurements, the action level at which one should consider to correct the monitor preset can be set equal to the uncertainty of the detector system. A number of authors derive action levels of around ±6-7% from their examinations, above which a correction of treatment parameters should be considered [86, 113, 115].

For measurements in depth of tissue, often larger deviations from the expected dose are found than the uncertainties stated for the different detectors (which range between 1.5 and 5%). Mean deviations are typically shifted by a few per cent from expected dose, standard deviations range between 3.5% and 9.9%, and often single deviations are substantially larger than the detector uncertainty (outliers). The authors often explain the deviations by inexact positioning of the detector, miscalculation of monitor units (incorrect determination of target depth?) or increased absorption of radiation from bleeding. Severgnini et al. [107] report measurements with larger pieces of radiochromic film which were placed on top and below shielding disks used in breast treatment with electrons. By visual inspection of the films they can detect misplacements of the disk or misalignments of applicator and target, and by quantitative analysis find dose reductions by 30% in 2 patients, possibly caused by incorrect determination of target thickness, additional absorption by blood or incorrect matching of applicator and target. The assumption of errors in set-up or detector positioning is supported by two publications which report smaller deviations for entrance dose than for dose in depth [115, 119]). López -Tarjuelo et al. [118] develop a model to calculate probability density distributions and confidence intervals for the dose measured with MOSFET and radiochromic film detectors from the convolution of a probability distribution of detector response with the expected dose distribution (similar to the convolutions used in small field dosimetry). A measured dose deviation exceeding the confidence interval could then be considered to lie beyond an action level. In 30 measurements of dose to the tumor bed of 29 patients (delivered with electrons from an Elekta Precise accelerator) they find confidence interval widths between 8.6% and 14.7% around the expected dose level of 90% PDD. Using these widths as action level they find 37% outliers. For dose measurements in depth, IVD is apparently very uncertain, and it is questionable whether one can define meaningful action levels.

#### Controlled procedures for placement of in-vivo dosimeters

To reduce uncertainties in the in-vivo dosimetry set-up, controlled institution-specific procedures should be developed, e.g. to pre-plan IVD, and to instruct and train the involved (sterile) personnel how to correctly place and fix the detectors. Imaging of detector position, e.g. with ultrasound should help assigning detector position to measurement results. Publications of reliable procedures reducing uncertainty would be of help.

### Treatment planning, in-room imaging

Despite the named uncertainties in detector response and positioning, the results of in-vivo dosimetry indicate that that deviations from intended dose are present in IORT. Since, with exemption of a few in-house developed systems, no real time treatment planning for IORT is available, treatment is generally based on the concept that the dose distributions from an isodose atlas measured in a simplified set-up with the applicator positioned flat on a body of water (or a closely adherent body of water for the spherical applicators used in kV IORT) also apply in the patient. This is clearly not true. Gaps between applicator and tissue as well as the non-flat surface change the distance from the source. Collection of blood and other liquids on top of the tissue changes the target depth. Heterogeneities inside and adjacent to the radiation field, e.g. bones, air gaps or also shielding materials change radiation absorption and also scatter contributions. Additionally, the shape, dimensions and also the position of the target in the beam are not documented and often not completely known. The key developments of recording and planning therapy on images which lead to the major improvements of radiotherapy in roughly the last 35 years have practically not taken place in IORT. These uncertainties are usually acceptable in the classical approach of IORT to treat a thin superficial layer of target tissue, however can lead to significant errors when extended (thick) target volumes are treated which cannot be seen in total by surgeon and radiation oncologist e.g. in breast IORT.

#### In-room imaging in IORT

Major reasons for the lag in development of planning systems for IORT are the unsolved difficulties in installing useful in-room imaging in the OR. Devices providing the three-dimensional images used in modern treatment planning interfere with the limited space in an OR, consume additional operation time, and are difficult to position conserving the sterile surgical set-up. The patient often has an unfavorable and space-consuming position which makes placement of the imaging device and correct adjustment in relation to the target difficult. A sometimes unavoidable problem of intraoperative imaging is the image artifacts caused by metallic surgical tools and table parts. Replacing them by non-metallic materials is often not possible. Additionally, in-room imaging causes installation costs which are difficult to justify for a limited IORT program usually treating only a small number of patients per week. A strategy to improve the cost-efficiency of imaging devices may be to plan for special ORs which provide 3D-imaging for multiple purposes together with IORT.

**Fig. 8:**
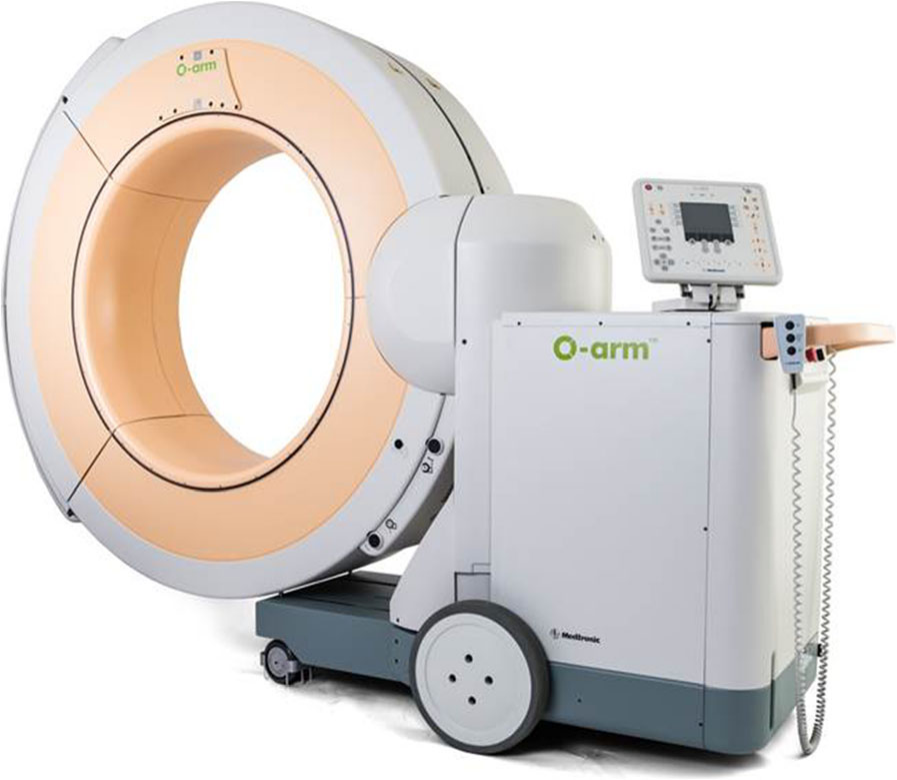
The O-arm mobile surgical cone beam CT imaging device (Medtronic GmbH, Earl-Bakken-Platz 1,40670 Meerbusch, Germany)

**Fig. 9:**
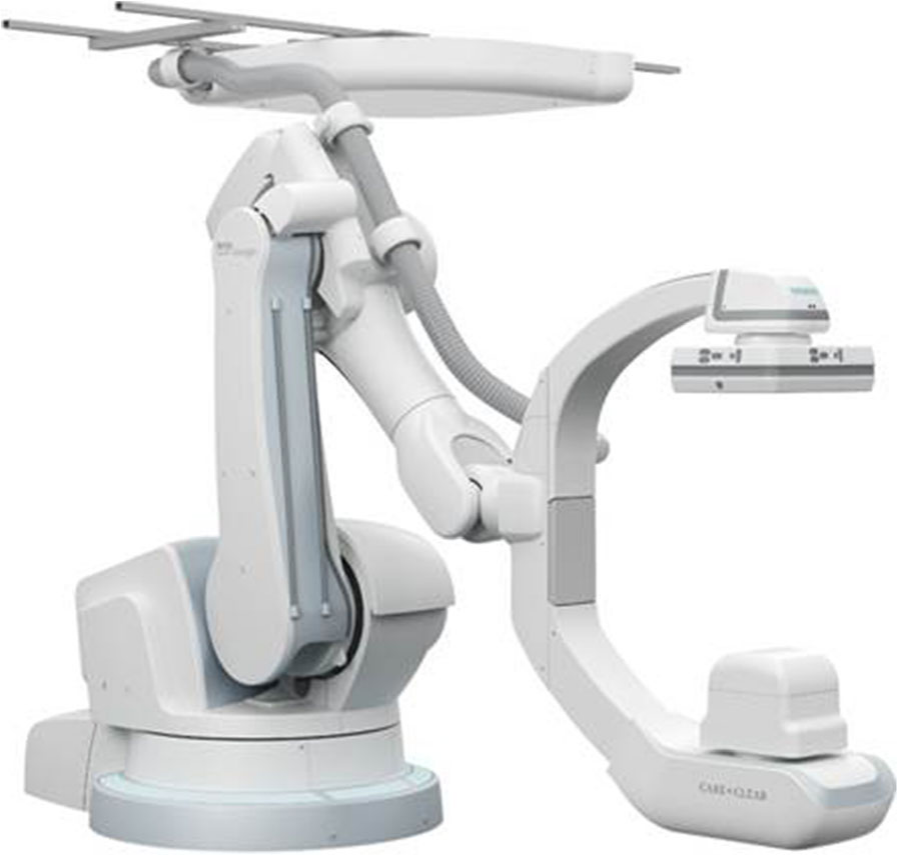
The robot-mounted flat panel surgical imaging device Siemens Artis zeego (Siemens Healthcare GmbH, Henkestr. 127, 91052 Erlangen, Germany)

**Fig. 10:**
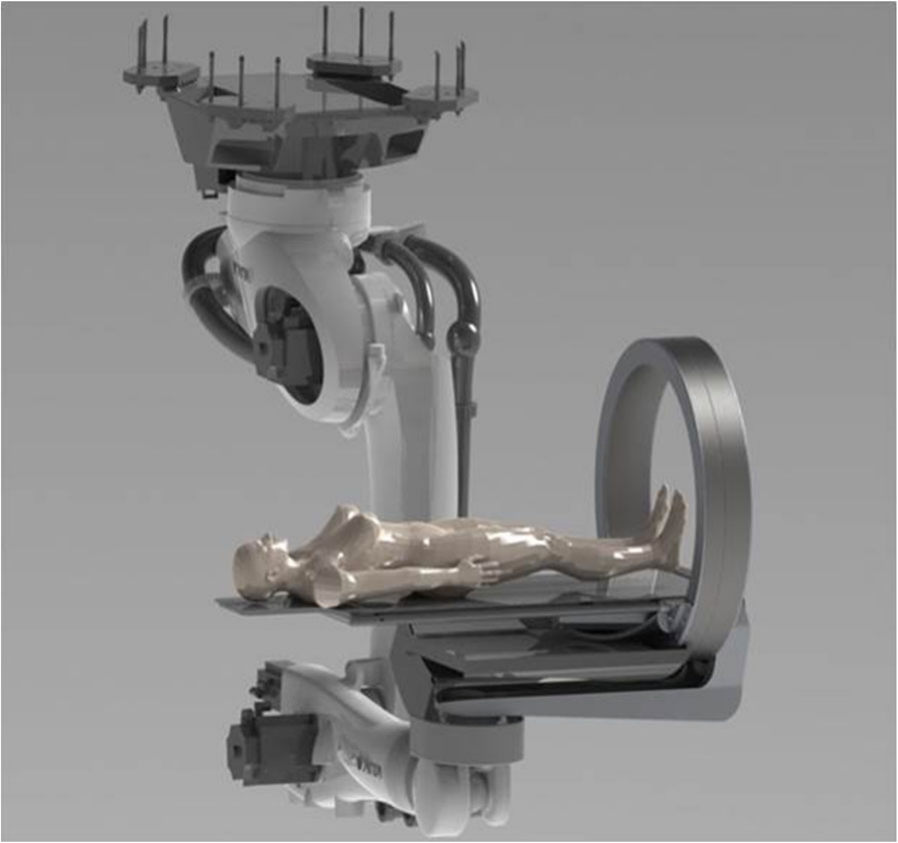
The PAIR patient alignment imaging ring (medPhoton G.m.b.H., Müllner Hauptstraße 48, 5020 Salzburg, Austria)

Although CT on rails technique is becoming present in radiotherapy vaults [124, 125] only one facility (in Klagenfurt, Austria) was found in this review providing this service in an IORT room. For In-OR imaging cone-beam CT (CBCT) facilities on mobile C-arm surgical fluoroscopy units have been developed [126], however these can often only reconstruct a small image volume which is difficult to position around the IORT treatment volume. Easier positioning is possible with the O-arm CBCT (Fig. 8). Larger reconstruction volumes and easier positioning in the OR setup are provided with newer C-arms which combine large flat panel detectors with robotic positioning (Fig. 9). A promising development is the PAIR system in development by the Salzburg Paracelsus Medical University in cooperation with MedAustron (Fig. 10) [127, 128]. The patient couch is mounted on a ceiling-mounted robot arm together with a large bore (60cm) sliding imaging ring. After loading (or possibly also operating) the patient on the couch, the ring is moved over the patient and acquires CBCT images with a large flat panel detector. Patient dose can be reduced by image reconstruction from only a limited part of the field of view. From the loading position the patient is moved with the robotic arm to an irradiation position under the gantry of an accelerator without repositioning on the couch.

An issue in using CBCT images for radiotherapy treatment planning is the larger uncertainty in the assignment of electron densities to CT-numbers [129] which is larger than for conventional fan-beam CT. This can result from different image acquisition parameters such as voltage settings but also depends on the different scatter contribution from different scanned volumes in different parts of the body [130]. Scatter causes a larger background of noise and streak and cupping artifacts in reconstruction [131, 132]. Strategies to reduce calculation errors from incorrect densities may be the use of multiple calibration curves for different sites [130, 133], manual assignment of densities to segmented tissue regions, mapping electron densities form conventional CT to CBCT [134] or matching the applicator and target position from an in-room CBCT to a previously acquired conventional CT and using this for planning.

#### Treatment planning systems for IORT

So far, there is no commercial treatment planning system which uses in-room imaging. The single existing system for IORT planning [135] combines a surgical navigation system with elaborate tools for volume rendering of CT images with the possibilities to simulate a surgical cavity, define an applicator in position and angle, and calculate the dose distribution. This can be used to pre-plan the surgical IORT procedure or to reconstruct the dose distribution, usually on an independent CT study. For electron dose calculation the system uses a pencil beam model. A Monte Carlo based model is in development using phase space files of the electron fluence at the applicator end as source model. In a second line of development the system a hybrid Monte Carlo model (“dose painting”) [136] is used to calculate dose distributions for low energy X-rays from Intrabeam applicators. Additionally the system provides reporting tools and can be connected to verify and record systems including DICOM RT export. In summary, further developments in IORT in-room imaging and in treatment planning are needed.

### Reporting IORT

In lack of a planning system and computerized tools for documentation, manual records of IORT are essential. These should include all information necessary to reconstruct the anatomical site and distribution of dose at a later time, e.g. to plan additional external beam radiotherapy or to correlate a recurrence with the first treatment. Therefore the record should include anatomical sketches showing the treatment site and possible shielded areas, applicator size and location including the angle of beam incidence, beam energy, information on bolus, gaps or any circumstance altering the dose distribution. For later evaluation and pooled analysis, participation in IORT registries is helpful.

### Uncertainties and deviations from intended dose in IORT

The possible deviation of the dose applied in IORT from the desired value can be caused in part from the uncertainty in its physical determination. This is composed of the uncertainty in the reference dosimetry (calibration) with which the absolute output of the irradiator is measured, the uncertainty in the measurement (and recording etc.) of the dose distributions and the reproducibility of the output between a calibration and a treatment or repeated measurement at a later time. If one restricts this consideration to absorbed dose to water, no uncertainties in the transformation of dose to other materials or biological doses occur. An additional source of uncertainty stems from possible deviations from the intended set-up. If the detector is placed correctly, this component, together with the reproducibility of the output and the uncertainty of the measurement is the major source of the dose deviations seen in in-vivo dosimetry. A third source of dose deviation is caused by the inhomogeneous dose distributions applied in IORT. Although this component is not an uncertainty it also contributes to the dose differences which determine the different outcome of two treatments (by the same or by different techniques). All of these components are considered in the following discussion in order to estimate a general number for the probable dose deviation, and to identify the physical means with which the deviations can be reduced. Additional file 2 compiles/summarizes the deviations found in this review for IORT with electrons and for kV X-rays and calculates the range of probable total dose deviations (Dev_total_) by summing the components in quadrature. Additional file 2 summarizes the uncertainties and dose deviations described below in detail.

#### IORT with electrons

Due to the non-reference conditions, uncertainty in the calibration of IORT electron beams is slightly larger than the uncertainty of 2.1% estimated by IAEA TRS 398 [63] for external beam accelerators. For the output of beveled applicators an additional (estimated) uncertainty of around 2-5% should be added in quadrature due to the uncertainty in finding the correct clinical axis and due to the asymmetric and deformed dose distributions of these applicators. For machines with high doses per pulse an additional uncertainty of 0.5-3% (taken as the variation of the recombination corrections for different ionization chambers) in the correction of recombination losses must be added, depending on pulse dose and method of correction. The total dosimetric uncertainty in reference dosimetry can therefore be estimated to be 2.2%-3.7% for straight and 2.9%-6.2% for beveled applicators. The uncertainty due to output instability lies around 2% [81] for stable and between 3.5% [106] and 9.9% [86] (standard deviation of entrance dose measurements) for some instable IORT accelerators. In Additional file 2, the uncertainty due to unstable output is not included in the calculation of the total deviation because it is part of the dose deviations found in in-vivo dosimetry (IVD). The standard deviations of the IVD measurements are also taken as measure for the uncertainty caused by set-up errors such as incorrect assessment of target size, incomplete coverage of the target due to applicator misalignment or incorrect beam entrance angle. These uncertainties are usually not present in treatments of targets of only a few millimeters thickness, however gaps or liquid collection under the applicator can also occur here. Neglecting the need for a 1-2cm larger applicator diameter in breast treatments can lead to target underdoses of 50% and more. This effect is not considered in the calculation of the total deviation since it can easily be avoided. The components of “uncertainty” named above are added in quadrature to arrive at a total probable deviation (Dev_total_) of the applied from the intended dose between 4.1% and 11.7%. Since most components are taken as one standard deviation of the applying dose variations, one can estimate that the applied dose will lie in an interval of ± (Dev_total_) around the intended dose with a probability of 68%.

A larger spread of dose deviation is caused by the fact that the treatment dose is prescribed to the 90% value of the inhomogeneous dose distribution produced by the electron beam. This leads to a intentionally planned dose variation in the target between 100% and 111% of the prescription value. This shall be called the variation of *intended dose*. It is this range of intended IORT dose which e.g. can be compared to the doses or the dose-effects of other methods. Since IORT with electrons places the prescription dose at the lower end of the range, one can expect that dose-effects will probably be larger than those assigned to the prescription dose. When considering uncertainties, the total deviations Dev_total_ will add linearly to the intended dose.

#### kV-IORT systems

The uncertainties reported for the calibrations of kV IORT systems by different investigators range between 5.3% [90] and 10.8% [17–19]. Leaving aside the unexplained uncertainties in the Intrabeam calibration but considering the differences of 4.8-8.8% between depth dose curves measured by the manufacturer and independently, one may estimate the uncertainty of dose delivery by X-ray systems of the Intrabeam and Papillon type to be around 10%. Output stability for the Intrabeam is continuously measured during treatment, so that it should hardly contribute to uncertainty. In a comparison of four PRS sources, Armoogum et al. [18] report a reproducibility of the internal radiation monitor within 0.23% with differences of the mean dose at differences angles within 0.49%. Uncertainties in the set-up can occur by missing adherence of the tissue to the applicator surface. For a 40mm diameter applicator, an air gap of 1mm between the applicator surface and the tissue reduces the dose to the underlying tissues by 9% (calculated with the inverse square law). If the gap is filled with liquid, the additional absorption will increase the dose reduction to 14% (as read from the Zeiss depth dose curves). For a 2mm gap the dose reductions will be 17% for air and 26% for a liquid-filled gap. The uncertainties in calibration and dose distribution add to a total uncertainty of 7.2-13.4%. If gaps between applicator and target tissue occur, the total deviations can rise to between10.5%-15% and 20.1%-28.2%.

Due to the short distance the source and the strong absorption of low energy X-rays in tissue, the dose variation in the target is very large: in a 10mm shell of target tissue dose decreases to 34% of surface dose for a 5cm applicator and to25% for a 3.5cm applicator. At 20 mm distance from the surface, the dose has dropped to 15% (5cm applicator) or 10% (3.5cm applicator). The remaining total dose deviations will add to the levels of intended dose given by these variations. To compare the variation of intended dose for breast treatments with kV X-ray systems one must acknowledge that the prescription dose at the applicator surface is about twice the dose prescribed in electron treatments. This implies that means that, for breast IORT, a dose distribution of between 111% and 100% of prescription dose with an uncertainty of around ±12% with electrons would compare to a dose distribution between 200% and 66-75% (for 10mm target thickness or 20%-30% for 20mm) with an uncertainty between 10.5% and 28.2% using kV X-rays. Radiobiological models by Herskind et al. [137–139] attempt to explain why the kV dose distributions may give comparable treatment results to electrons, however a proof of equivalence can only be given by the final results of the Targit [22–24] and possibly additional trials.

In summary, while the uncertainties in the dosimetry of IORT are similar to those in external beam therapy (when the issues in the Intrabeam calibration are clarified), the possible unexpected deviations from intended dose due to set-up uncertainties such as incorrect assessment of target dimensions, gaps, blood pooling, etc. reported for IORT are around 4-12% for electrons and 10%-28% for kV X-rays. The set-up imponderabilia are measured by in-vivo dosimetry as 5-10% with frequent larger outliers up to 30%. The in-vivo measurements include the possible deviations in calibration and output stability. This group of dose deviations can possibly be reduced by physical improvements in dosimetry. The uncertainties in target assessment and applicator placement could be reduced by on-line imaging and treatment planning and therefore give reason for further development of appropriate tools.

For breast treatments, the uncertainties add on to deviations from intended prescription dose of 11% for electrons and 25-34% (or70-80%) for kV X-rays inherent to the dose gradients of the radiation, making the dose-effect relations and outcomes of these treatments difficult to predict or compare. This uncertainty gives need to prove the efficacy of IORT in clinical trials, however is probably outweighed by the obvious advantages of increasing dose while reducing of dose to neighboring healthy tissues, reduction of geographical miss, early application of treatment before further growth of remaining tumor cells can occur, and possible biological advantages of irradiation with single high doses.

## Conclusions

Although IORT in its present form has been used since over 40 years, some basic requirements to ensure quality and reproducibility of treatment remain in need of further development. Issues found in this review include better definition and documentation of IORT targets as well as the need to further develop treatment planning and reporting. Other uncertainties and surgical limitations in the application such as bleeding may be more difficult to exclude.

The possibilities to reduce the uncertainties in the calibration and dosimetry of treatment with kV X-rays should further investigated. In total one may assume a typical uncertainty of ±10-15% for IORT procedures. To assess the dose response of IORT one must additionally consider the dose inhomogeneity inherent to the different technologies. Probably the present level of uncertainty is acceptable if one considers the potential benefits of IORT for the patient. However, effort should be taken to reduce uncertainty and the present uncertainty must be considered if one approaches tolerance doses.

IORT can help improve radiation therapy by sterilizing cells remaining in the tumor bed after surgical resection (“reducing surgical margins”), by allowing an advanced boost before further tumor growth, by avoiding geographical miss and in general by escalating the dose to the tumor. A further advantage which is difficult to measure is the mutual education of the involved disciplines in judging the situation after tumor resection and the possibilities of adapting radiotherapy to it. These potential benefits from IORT have been difficult to prove, since randomized trials are scarce. However pooled analysis has shown improvements by IORT, as demonstrated in the reviews in this journal. Uncertainties in dose, dose distribution and dose-effect-relation, in documentation of the treatment and the exact treatment site add to the difficulty in proving evidence of therapeutic benefit. The reduction of these uncertainties may help improve the quality of IORT, and help provide better data to prove evidence of the therapeutic benefit of IORT.

## Additional files

Additional file 1: Compilation of publications on in-vivo dosimetry for IORT. The publications are sorted by detector type and irradiation device. Deviations from expected dose found in the publication and, when available, associated action levels concluded by the authors are highlighted. (PDF 343 kb)
Additional file 2: Uncertainties and dose deviations in IORT. (PDF 234 kb)

## Abbreviations

AAPM: American Association of Physicists in Medicine
AAPM TG: AAPM Task Group
APBI: Accelerated partial breast irradiation
AV: Acceleration voltage
CBCT: Cone beam CT
DVH: Dose-volume histogram
EBT: Type of radiochromic film
HVL: Half value layer
IC: Ionization chamber
IORT: Intraoperative radiation therapy
IPSA: Inverse optimization with simulated annealing
IVD: In-vivo dosimetry
Linac: linear accelerator
MOSFET: Metal-oxide-semiconductor field-effect transistor
NIST: U.S. National Institute of Standards and Technology
OSLD: Optically stimulated luminescence detector
PAICH: Zeiss QA tool: Probe Adjustment and Ionization Chamber Holder
PDA: Zeiss QA tool: Photodiode Array
PMMA: Polymethylmethylacrelate = Perspex
PRS: Photon Radiosurgery System
PTB: Physikalisch-Technische Bundesanstalt, German national standards laboratory
QA: Quality assurance
TLD: Thermoluminescence dosimeter
TVA: Two voltage analysis
XRS: X-ray source (abbreviation used by Zeiss)
