# Supplementary material for: Present state and issues in IORT Physics
Source: Radiat Oncol. 2017 Jan 27;12:37. doi: 10.1186/s13014-016-0754-z (PMC5307769; doi:10.1186/s13014-016-0754-z)
Supplement: Additional file 1: — Compilation of publications on in-vivo dosimetry for IORT. The publications are sorted by detector type and irradiation device. Deviations from expected dose found in the publication and, when available, associated action levels concluded by the authors are highlighted. (PDF 343 kb) [file 13014_2016_754_MOESM1_ESM.pdf]

Publication	Detector	Treated site	Measured quantity	Treatment device	No. Patients or measurements	Deviation from expected dose min	max	mean	median	SD or range	Uncertainty stated by authors	Action level	Other results
Agostinelli 2012 (86)	MOSFET	breast	expected target dose	Liac	91	-14,0%	25,0%	9,1%		9,9%	5,0%	±6%	
			with COR-related correction<br>of output instability		45	-11,0%	22,0%	7,6%		9,5%			
Ciocca 2006 (105)	MOSFET	breast	entrance dose	Novac 7	45	-7,6%	10,0%	0,6%		3,5%	3,6%	±6%	
Soriani 2007 (113)	MOSFET	prostate	dose / setup verification<br>under beveled applicator	Novac 7	12	-10,7%	10,0%	0,6%	0,4%	7,6%	2,9%		perturbation by MOSFET + catheter < 2%
Consorti 2005 (114)	MOSFET	definitive breast	target exit dose	Novac 7	12	-7,0%	5,0%	1,1%	2,8%	±5%	1,7-2,1%		attenuation by catheter + dosimeter<br>1.5-20% at low electron energies
		breast boost / pancreas	target entrance dose<br><br>0° applicators		7	-1,6%	11,6%	3,9%	3,4%		angular dependence<br>>10% (45°)<br>20% (90°)<br>total uncertainty ±3,5%	7%	
Bloemen-van Gurp et al. (123)	MOSFET	non-IORT	entrance dose	general purpose accelerator	40			-0,7%		2,9%	angular dependence negligible < 45°<br><15% (60°)<br>24% (90°)		corrections applied for SSD, field size and shape
Lopez-Tarjuelo 2014 (115)	MOSFET<br>radiochrom. film	vaious	dose to tumor bed	general pupose accelerator	30 MOS	-22,0%	6,6%	-6,5%	-6,1%	6,5%	2,2%		
			expected dose=100%		29 film<br>27 pats.	-17,2%	3,6%	-4,0%	-4,7%	5,5%	2,8%		
Lopez-Tarjuelo 2016 (116)	MOSFET<br>radiochrom. film	various<br>37% breast<br>29% colorectal	dose to tumot bed	Elekta Precise	40 MOS	-22,0%	11,6%	-6,2%	-7,2%	6,7%	1,5%		
			expected dose=100%		42 film<br>45 pats.	-28,0%	23,4%	-2,1%	-1,9%	9,0%			
Lopez-Tarjuelo 2016a (117)	MOSFET<br>radiochrom. film	various	dose to tumor bed<br>expected dose=100%<br>assesment action level		30 measurements			4,0%			2-2,2% MOSFET<br>2,7% film		width of confidence interval between 8.6%and 14.7%<br>in relation to expected dose level of
Ciocca 2003 (105)	radiochrom. film	breast	entrance dose	Novac 7	35	-9,9%	9,9%	1.8%		4,7%			
Krengli 2010 (118)	radiochrom. film	prostate	dose to rectum	Mobetron	38	n/a	n/a						
Severgnini 2014 (106)	radiochrom.	breast	dose above/	Mobetron	37	-10,0%	8,0%	-2,8%	-1,0%				information on position of

	film		under shielding disk			max -39% from misplacement ± miscalculation					shielding disk and<br>alignment of applicator
Tabarelli de Fatis et al. (119)	radiochrom Film	breast	entrance dose<br>dose in depth<br>alignment of beam and target	Liac	63		0.6%<br>7.2%	5%<br>7%			deviation also due to backscatter from attenuator plate
Avanzo 2012 (95)	radiochrom. film	breast	dose at applicator surface<br>(in surgical cavity)<br>skin dose<br>dose under tungsten shield	Intrabeam	23 pats.	average deviation of dose at appl. surface:<br>3.5cm appl.<br>4.0cm appl.<br>4.5cm appl.<br>5.0cm appl.	 -27,6%<br>-19,9%<br>-11,9%<br>-10,4%		2,80%		dose averaging on flat film in steep dose gradient around spherical applicator?
Price 2013 (96)	OSL D	breast	dose at applicator surface<br>(in surgical cavity)<br>skin dose	Intrabeam	20 pats.	average deviation of dose at appl. surface:<br>3.0 cm appl. (3 measurements)<br>4.5 cm appl. (0)<br>4.0 cm appl. (3)<br>4.5 cm appl. (3)<br>5.0cm appl. (3)	 -1,0%<br><br>-21,8%<br>-30,3%<br>-13,6%		7% OSLD uncertainty<br>at appl. Surface<br>17% at skin		reduced absorption of -20.5 % to -4.1% by OSLD + housing, OSLD alone +5.9% absorption<br>+5.9% absorption assumed
	radiochrom. film					3.0 cm appl. (3)<br>3.5 cm appl. (4)<br>4.0cm appl. (8)<br>4.5cm appl. (5)<br>5.0 cm appl. (0)	6,3%<br>-7,3%<br>-11,0%<br>-16,6%		±8% assumed film uncertainty		2.6% to 8.5% absorption quoted for film<br>8,5% absorption assumed
Fogg 2010 (120)	TLD	breast	skin dose	Intrabeam	57	n/a n/a			17% total		
Eaton 2012 (108)	TLD	breast	skin dose	Intrabeam	72	n/a n/a			8% energy response<br>5% lack of backscatter<br>3-9% energy response		dose at phantom surface:<br>5% dose reduction with 1 sheet of tungsten rubber as backscatterer<br>2% doses enhancement with 2 sheets of tungten rubber<br>4% dose reduction with 1 cm Plastic<br>2 sheets tungten
