# Supplementary material for: Present state and issues in IORT Physics
Source: Radiat Oncol. 2017 Jan 27;12:37. doi: 10.1186/s13014-016-0754-z (PMC5307769; doi:10.1186/s13014-016-0754-z)
Supplement: Additional file 2: — Uncertainties and dose deviations in IORT. (PDF 234 kb) [file 13014_2016_754_MOESM2_ESM.pdf]

Publication	Detector	Treated site	Measured quantity	Treatment device	No. Patients<br>or measurements	Deviation from expected dose<br>min	 max	 mean	 median	SD<br>or range	Uncertainty<br>stated by authors	Action level	Other results
Agostinelli 2012 (86)	MOSFET	breast	expected target dose<br>with COR-related correction<br>of output instability	Liac	91<br>45	-14,0%<br>-11,0%	25,0%<br>22,0%	9,1%<br>7,6%		9,9%<br>9,5%	5,0%	±6%	
Ciocca 2006 (105)	MOSFET	breast	entrance dose	Novac 7	45	-7,6%	10,0%	0,6%		3,5%	3,6%	±6%	
Soriani 2007 (113)	MOSFET	prostate	dose / setup verification<br>under beveled applicator	Novac 7	12	-10,7%	10,0%	0,6%	0,4%	7,6%	2,9%		perturbation by MOSFET + catheter < 2%
Consorti 2005 (114)	MOSFET	definitive breast<br>breast boost /<br>pancreas	target exit dose<br>target entrance dose<br>0° applicators	Novac 7	12<br>7	-7,0%<br>-1,6%	5,0%<br>11,6%	1,1%<br>3,9%	2,8%<br>3,4%	±5%	1,7-2,1%<br>angular dependence<br>>10% (45°)<br>20% (90°)<br>total uncertainty ±3,5%	7%	attenuation by catheter + dosimeter<br>1.5-20% at low electron energies
Bloemen-van Gurp et al. (123)	MOSFET	non-IORT	entrance dose	general purpose accelerator	40			-0,7%		2,9%	angular dependence<br>negligible < 45°<br><15% (60°)<br>24% (90°)		corrections applied for SSD, field size and shape
Lopez-Tarjuelo 2014 (115)	MOSFET<br>radiochrom. film	vaious	dose to tumor bed<br>expected dose=100%	general pupose<br>accelerator	30 MOS<br>29 film<br>27 pats.	-22,0%<br>-17,2%	6,6%<br>3,6%	-6,5%<br>-4,0%	-6,1%<br>-4,7%	6,5%<br>5,5%	2,2%<br>2,8%		
Lopez-Tarjuelo 2016 (116)	MOSFET<br>radiochrom. film	various<br>37% breast<br>29% colorectal	dose to tumot bed<br>expected dose=100%	Elekta Precise	40 MOS<br>42 film<br>45 pats.	-22,0%<br>-28,0%	11,6%<br>23,4%	-6,2%<br>-2,1%	-7,2%<br>-1,9%	6,7%<br>9,0%	1,5%		
Lopez-Tarjuelo 2016a (117)	MOSFET<br>radiochrom. film	various	dose to tumor bed<br>expected dose=100%<br>assesment action level		30 measurements			4,0%			2-2,2% MOSFET<br>2,7% film		width of confidence interval between 8.6%and 14.7%<br>in relation to expected dose level of 90% (p=0.05)
Ciocca 2003 (105)	radiochrom. film	breast	entrance dose	Novac 7	35	-9,9%	9,9%	1.8%		4,7%			
Krengli 2010 (118)	radiochrom. film	prostate	dose to rectum	Mobetron	38	n/a	n/a						
Severgnini 2014 (106)	radiochrom. film	breast	dose above/<br>under shielding disk	Mobetron	37	-10,0%<br>max -39% from misplacement ± miscalculation	8,0%	-2,8%	-1,0%				information on position of shielding disk and alignment of applicator

Tabarelli de Fatis et al. (119)	radiochrom Film	breast	entrance dose<br>dose in depth<br>alignment of beam and target	Liac	63	0.6%<br>7.2%	5%<br>7%			deviation also due to backscatter from attenuator plate
Avanzo 2012 (95)	radiochrom. film	breast	dose at applicator surface<br>(in surgical cavity)<br>skin dose<br>dose under tungsten shield	Intrabeam	23 pats.	average deviation of dose at appl. surface:<br>3.5cm appl. -27,6%<br>4.0cm appl. -19,9%<br>4.5cm appl. -11,9%<br>5.0cm appl. -10,4%		2,80%		dose averaging on flat film in steep dose gradient around spherical applicator?
Price 2013 (96)	OSL D	breast	dose at applicator surface<br>(in surgical cavity)<br>skin dose	Intrabeam	20 pats.	average deviation of dose at appl. surface:<br>3.0 cm appl. (3 measurements) -1,0%<br>4.5 cm appl. (0)<br>4.0 cm appl. (3) -21,8%<br>4.5 cm appl. (3) -30,3%<br>5.0cm appl. (3) -13,6%		7% OSLD uncertainty<br>at appl. Surface<br>17% at skin		reduced absorption of -20.5 % to -4.1% by OSLD + housing, OSLD alone +5.9% absorption<br>+5.9% absorption assumed
	radiochrom. film					3.0 cm appl. (3) 6,3%<br>3.5 cm appl. (4) -7,3%<br>4.0cm appl. (8) -11,0%<br>4.5cm appl. (5) -16,6%<br>5.0 cm appl. (0)		±8% assumed film uncertainty		2.6% to 8.5% absorption quoted for film<br>8,5% absorption assumed
Fogg 2010 (120)	TLD	breast	skin dose	Intrabeam	57	n/a n/a		17% total		
Eaton 2012 (108)	TLD	breast	skin dose	Intrabeam	72	n/a n/a		8% energy response<br>5% lack of backscatter<br>3-9% energy response		dose at phantom surface:<br>5% dose reduction with 1 sheet of tungsten rubber as backscatterer<br>2% doses enhancement with 2 sheets of tungten rubber<br>4% dose reduction with 1 cm Plastic water + 2 sheets tungten
